# Using SCENTinel® to predict SARS-CoV-2 infection: insights from a community sample during dominance of Delta and Omicron variants

**DOI:** 10.3389/fpubh.2024.1322797

**Published:** 2024-04-10

**Authors:** Stephanie R. Hunter, Anne Zola, Emily Ho, Michael Kallen, Edith Adjei-Danquah, Chad Achenbach, G. Randy Smith, Richard Gershon, Danielle R. Reed, Benjamin Schalet, Valentina Parma, Pamela H. Dalton

**Affiliations:** ^1^Monell Chemical Senses Center, Philadelphia, PA, United States; ^2^Feinberg School of Medicine, Northwestern University, Chicago, IL, United States

**Keywords:** COVID, prediction, olfaction, anosmia, testing, hyposmia, pandemic

## Abstract

**Introduction:**

Based on a large body of previous research suggesting that smell loss was a predictor of COVID-19, we investigated the ability of SCENTinel®, a newly validated rapid olfactory test that assesses odor detection, intensity, and identification, to predict SARS-CoV-2 infection in a community sample.

**Methods:**

Between April 5, 2021, and July 5, 2022, 1,979 individuals took one SCENTinel® test, completed at least one physician-ordered SARS-CoV-2 PCR test, and endorsed a list of self-reported symptoms.

**Results:**

Among the of SCENTinel® subtests, the self-rated odor intensity score, especially when dichotomized using a previously established threshold, was the strongest predictor of SARS-CoV-2 infection. SCENTinel® had high specificity and negative predictive value, indicating that those who passed SCENTinel® likely did not have a SARS-CoV-2 infection. Predictability of the SCENTinel® performance was stronger when the SARS-CoV-2 Delta variant was dominant rather than when the SARS-CoV-2 Omicron variant was dominant. Additionally, SCENTinel® predicted SARS-CoV-2 positivity better than using a self-reported symptom checklist alone.

**Discussion:**

These results indicate that SCENTinel® is a rapid assessment tool that can be used for population-level screening to monitor abrupt changes in olfactory function, and to evaluate spread of viral infections like SARS-CoV-2 that often have smell loss as a symptom.

## Introduction

The prevalence of COVID-19 disease (diagnosed or confirmed by a positive SARS-CoV-2 reverse-transcriptase PCR or antigenic test result) has varied from 1.6 to 8.6% in the United States (1). Due to a delay in test development, a lack of consistent testing and/or reporting, as well as high false-negative results of initial PCR assays for SARS-CoV-2 (2, 3), the true prevalence of COVID-19 will likely remain unknown. Numbers of hospitalizations and deaths have been used in the past to understand COVID-19 prevalence and disease burden, but these metrics do not provide a complete picture of population-wide SARS-CoV-2 active infection and its spread, not least because they can easily miss individuals who are infected with SARS-CoV-2 yet who do not report relevant symptoms, that may be specific to CoV-2 infections (1, 4, 5). Identifying and isolating asymptomatic individuals are critical steps during a pandemic when tests, vaccines, and treatments are not widely available.

In the early days of the COVID-19 pandemic, fever, cough, and shortness of breath were identified as cardinal symptoms of SARS-CoV-2 infection (6). However, smell loss was soon discovered as a more sensitive and earlier predictor of active SARS-CoV-2 infection than these other cardinal symptoms (7–9). Early prevalence estimates, obtained when the Alpha and Delta SARS-CoV-2 variants were dominant, indicated that approximately 67% of those with COVID-19 lost their sense of smell during active infection (10). Yet, when new SARS-CoV-2 variants emerged in the population, they were associated with a reduced prevalence of smell loss (11). For instance, in those infected with the Omicron variant, the clinical profile is markedly different, and only about 20% of individuals lost their sense of smell (11–13).

Subtle and gradual changes in olfactory abilities, such as those occurring with aging, are not reliably self-reported (14–16). Even though smell loss with SARS-CoV-2 occurred suddenly and often in the early phase of the disease, a meta-analysis showed that directly testing an individual’s smell function reveals significantly more cases of smell loss compared to relying on self-reported smell status (10). This suggested that detecting SARS-CoV-2 infection at the population level utilizing a test of olfactory function, as opposed to tracking onset of fever, would provide a more accurate representation of active SARS-CoV-2 cases and their spread (7, 17–26).

Most commercially available smell tests are limited in their utility (e.g., in a research study, or specialized clinic, but not at point-of-care), because they are expensive, are time consuming, or require specialized training to be administered (27, 28), making them unsuitable for population-wide surveillance of smell function, like was needed during the COVID-19 pandemic (29). Of the varied olfactory dimensions—e.g., detection, intensity, and identification—most available smell tests measure only one: identification (28, 30), the olfactory function most prone to cultural and cognitive biases (31–33). We do not yet know which components of olfactory function are most impacted in individuals infected with SARS-CoV-2. If the loss of smell is complete (anosmia), no odor will be detected and other olfactory functions cannot be ascertained, yet if olfaction is diminished (hyposmia), an odor may be detected and correctly identified even though the odor’s intensity is reduced. Thus, multifunction tests are needed to accurately identify SARS-CoV-2-related olfactory changes.

SCENTinel® is a smell test developed to measure multiple dimensions of olfactory function (i.e., odor detection, intensity, and identification, summed into an overall score) within 2 min, making it a suitable point-of-care screening tool to assess smell loss (29, 34). We have previously reported its ability to discriminate between those with a normal sense of smell (i.e., normosmia) and those with anosmia, hyposmia, and parosmia (i.e., odor distortions from a known source) (29, 34).

The purpose of this study was to determine whether SCENTinel® could screen for active SARS-CoV-2 infection, as determined by the gold-standard PCR test. We hypothesized that (1) fewer participants who are SARS-CoV-2 positive (C19+) vs. negative (C19−) by PCR test result would meet the accuracy criteria of the SCENTinel® odor detection subtest, (2) the average rating participants provide for the SCENTinel® odor intensity subtest would be lower for C19+ than for C19− people, (3) fewer C19+ vs. C19− participants would meet the accuracy criteria of the SCENTinel® odor identification subtest, (4) fewer C19+ vs. C19− participants would meet the accuracy criteria of the SCENTinel® test overall score, and (5) the SCENTinel® test status alone or in conjunction with other available data (i.e., demographics, health history, medical records data, and self-reported symptoms) would correctly predict C19+ vs. C19− status.

## Materials and methods

### Participants

Participants in this cross-sectional study were a community sample who presented at three Northwestern Medicine COVID-19 testing clinics in Illinois (Glenview, Lake Forest, and downtown Chicago COVID testing sites) for a physician-ordered SARS-CoV-2 PCR test between April 5, 2021, and July 5, 2022. The population tested included both symptomatic and asymptomatic participants, such as people needing preoperative or travel clearance (Supplementary Table S1). A total of 1,979 participants with matched SCENTinel® and SARS-CoV-2 test results were included in the final analysis (see Table 1).

**Table 1:** Demographic information for the sample of participants included in the final analyses (*n* = 1979).

Characteristic	*n* (%)
Age, mean (SD)	50.17 (16.06)
Gender	
Man	712 (36.0)
Woman	1,212 (61.2)
Neither	6 (0.3)
Missing	49 (2.5)
Race	
White	1,491 (75.3)
Black	198 (10.0)
Asian	92 (4.6)
American Indian	5 (0.3)
Native Hawaiian	1 (0.1)
More than one	30 (1.5)
Missing	162 (8.2)
Preexisting condition (*n* = 1,301)	
Asthma	301 (15.2)
Chronic obstructive pulmonary disease	37 (1.9)
Diabetes mellitus	155 (7.8)
Renal insufficiency	166 (8.4)
Chronic liver disease	91 (4.6)
Hypertension	562 (28.4)
HIV/AIDS	112 (5.7)
Immunological disease	168 (8.5)
Disturbances of smell/taste	27 (1.4)
Cancer	731 (36.9)

Preexisting conditions were provided by the patients’ electronic medical records. All other characteristics were self-reported.

### SCENTinel®

The SCENTinel® test is a rapid, accurate, inexpensive, and self-administered screening test that combines assessments of three components of olfactory function: odor detection, odor intensity, and odor identification (29, 34). The SCENTinel® test card contains three boxes (A, B, or C) using Lift’nSmell technology (Scentisphere, Carmel, NY, United States). Two of the boxes are blank, and one contains an odor. The odor location (under box A, B, or C) and the identity of the odor were randomized. The SCENTinel® test card is accompanied by an online survey, which is accessed by scanning a QR code or entering a URL, both located on the SCENTinel® test card. The SCENTinel® test uses one of multiple odorants with high familiarity in the United States population (30, 35, 36). In this study, we used two versions of SCENTinel® (1.1 and 2.0), which differed only in the identity of target odors that could be on the test. The rest of the test and instructions were the same across the two SCENTinel® versions. On SCENTinel® 1.1, the target odors were flower, coffee, bubblegum, or caramel popcorn. On SCENTinel® 2.0, the odors were flower, bubblegum, orange, coffee, banana, strawberry, coconut, woody, or lemon. Note that each SCENTinel® test contains only one of these odors. Distractors used in the odor identification question were chosen to be distinct so that they could not be easily confused with the target odor (37). All odors were used in concentrations to be rated an 80 out of 100 for someone with a normal sense of smell, based on pilot testing. Further odorant information can be found in Supplementary Table S2. SCENTinel® 1.1 test cards were used from April 5 to August 20, 2021 (34), and SCENTinel® 2.0 test cards were used from August 31, 2021 to July 5, 2022.

### SARS-CoV-2 tests

All three Northwestern Medicine COVID-19 testing clinics collected nasopharyngeal specimens by trained providers (38). Diagnostic techniques that are based on viral RNA amplification such as PCR used to confirm SARS-CoV-2 diagnosis are often considered the gold standard (39). A review paper notes that PCR has almost perfect specificity but low sensitivity (3). Thus, analysis was limited to individuals who were tested using PCR, which accounted for the overwhelming majority (98.6%) of total SARS-CoV-2 tests collected. The two primary PCR tests used were Multiplex PCR-Roche Cobas 8800 and Multiplex PCR.

### Procedures

All procedures were approved by the University of Pennsylvania Institutional Review Board as the IRB of record (protocol no. 844425) and were conducted according to the principles expressed in the Declaration of Helsinki. The COVID-19 testing clinic staff handed out SCENTinel® test cards to all patients and requested their voluntary participation in a research study. All SCENTinel® test cards were given before the nasopharyngeal swab for the SARS-CoV-2 test was collected. Participants who elected to participate were instructed to follow the instructions on the SCENTinel® card. Participants scanned a QR code, or entered a URL into a web browser to access the online REDCap survey (40), where they provided consent using an approved online consent form. They then answered demographic information (name, date of birth, sex, and race), indicated whether they had a preexisting smell or taste disorder, and self-reported whether they had any of the following 12 symptoms in the past 48 h (check all that apply): scratchy throat, painful sore throat, cough (worse than usual if you have a baseline cough), runny nose, symptoms of fever or chills, temperature greater than 100.4°F or 38.0°C, muscle aches (worse than usual if you have baseline muscle aches), nausea, vomiting or diarrhea, shortness of breath, unable to taste or smell, red or painful eyes, or none of the above. On the SCENTinel® test card, participants were then instructed to lift and smell boxes A, B, and C, one at a time (see Supplementary Figures S1, S2) and then to (1) select which odor smelled the strongest (odor detection; guessing probability = 33%); (2) rate the intensity of the strongest odor (odor intensity) on a visual analog scale (VAS) from 0 (labeled as “no smell”) to 100 (labeled as “very strong smell”; there were no other labels on the VAS); and (3) select what the odor smelled like among four options (i.e., one correct response and three distractors; odor identification; guessing probability = 25%) and, if incorrect, try once again from the three remaining options (guessing probability = 33%). The SCENTinel® odor identification subtest was the only subtest that participants were given a second attempt. Given the self-administered nature of the test, participants could have smelled the odor multiple times before answering the questions. This was not explicitly discouraged or encouraged in the instructions. The SCENTinel® odor detection and odor identification task (both its first and second attempt) were scored as dichotomies (i.e., correct/incorrect); the odor intensity rating was collected as a continuous variable and then scored as a dichotomy (i.e., ≤20 = smell loss; ≥21 = acceptable smell function) (8, 34). The odor intensity cutoff of 20 was used based on a previous study that found that those who rated a moderate to strong odor <20 on a 1–100 scale was indicative of COVID-19 related smell loss (8, 34). As summarized in Table 2, the SCENTinel® overall score was calculated based on odor detection, odor intensity, and odor identification response pattern and assigned a pass/fail status. Participants completed the whole test, irrespective of the accuracy of their responses. No feedback on the accuracy of each subtest response was provided except for the feedback on the first odor identification attempt (4-alternative forced choice), if the response was incorrect. They were then given the opportunity to complete a three-alternative forced choice with the remaining options. No feedback was provided to the second odor identification attempt. If participants correctly detected the odor, rated the odor intensity ≥21, and correctly identified the odor on the first attempt, a message stating, “You got it right!” appeared at the end of the survey.

**Table 2:** SCENTinel® test final score algorithm and guessing probabilities.

Response pattern	*N*	SARS-CoV-2 status (*n*)		Detection	Intensity (range: 1–100)	Identification attempt		Outcome	Probability (chance outcome)
		Positive	Negative			First	Second		
1	1,364	83	1,281	Correct	≥ 21	Correct	NA	Pass	0.07
2	238	14	224	Correct	≥ 21	Incorrect	Correct	Pass	0.07
3	120	9	111	Correct	≥ 21	Incorrect	Incorrect	Pass	0.13
4	42	1	41	Incorrect	≥ 21	Correct	NA	Pass	0.13
5	5	2	3	Correct	≤ 20	Correct	NA	Fail	0.02
6	4	1	3	Correct	≤ 20	Incorrect	Correct	Fail	0.02
7	7	2	5	Correct	≤ 20	Incorrect	Incorrect	Fail	0.03
8	0	0	0	Incorrect	≤ 20	Correct	NA	Fail	0.03
9	41	3	38	Incorrect	≥ 21	Incorrect	Correct	Fail	0.13
10	2	0	2	Incorrect	≤ 20	Incorrect	Correct	Fail	0.03
11	125	5	120	Incorrect	≥ 21	Incorrect	Incorrect	Fail	0.26
12	6	3	3	Incorrect	≤ 20	Incorrect	Incorrect	Fail	0.07

NA, Not applicable. We acknowledge that some participants may have selected a response by error, and this should not screen them as having a smell disorder if they answered other subtests accurately. Thus, in Response pattern 4, participants could still pass the test even if they answered odor detection incorrectly.

SARS-CoV-2 test results were stored in an electronic data warehouse (EDW) that integrated inpatient and outpatient records across Northwestern Medicine healthcare system practice settings. EDW records included the time and date of release of the patient’s SARS-CoV-2 result, the type of test (e.g., PCR vs. nucleic acid amplification tests), test result (positive, negative, or undetermined for SARS-CoV-2 infection), and comorbidities based on the International Classification of Diseases 9/10 codes. The EDW was used to match SARS-CoV-2 PCR test results with SCENTinel® results using participants’ first name, last name, and date of birth, which were self-reported in the SCENTinel® survey. If the first attempt to match SCENTinel® test results to EDW records was unsuccessful, we cleaned the reported first name, last name, and date of birth to improve matching. For example, we removed any numbers from name fields, removed suffixes or apostrophes, and changed date-of-birth years starting with “91″ to “19″ (see Appendix S1).

**Figure 1:**
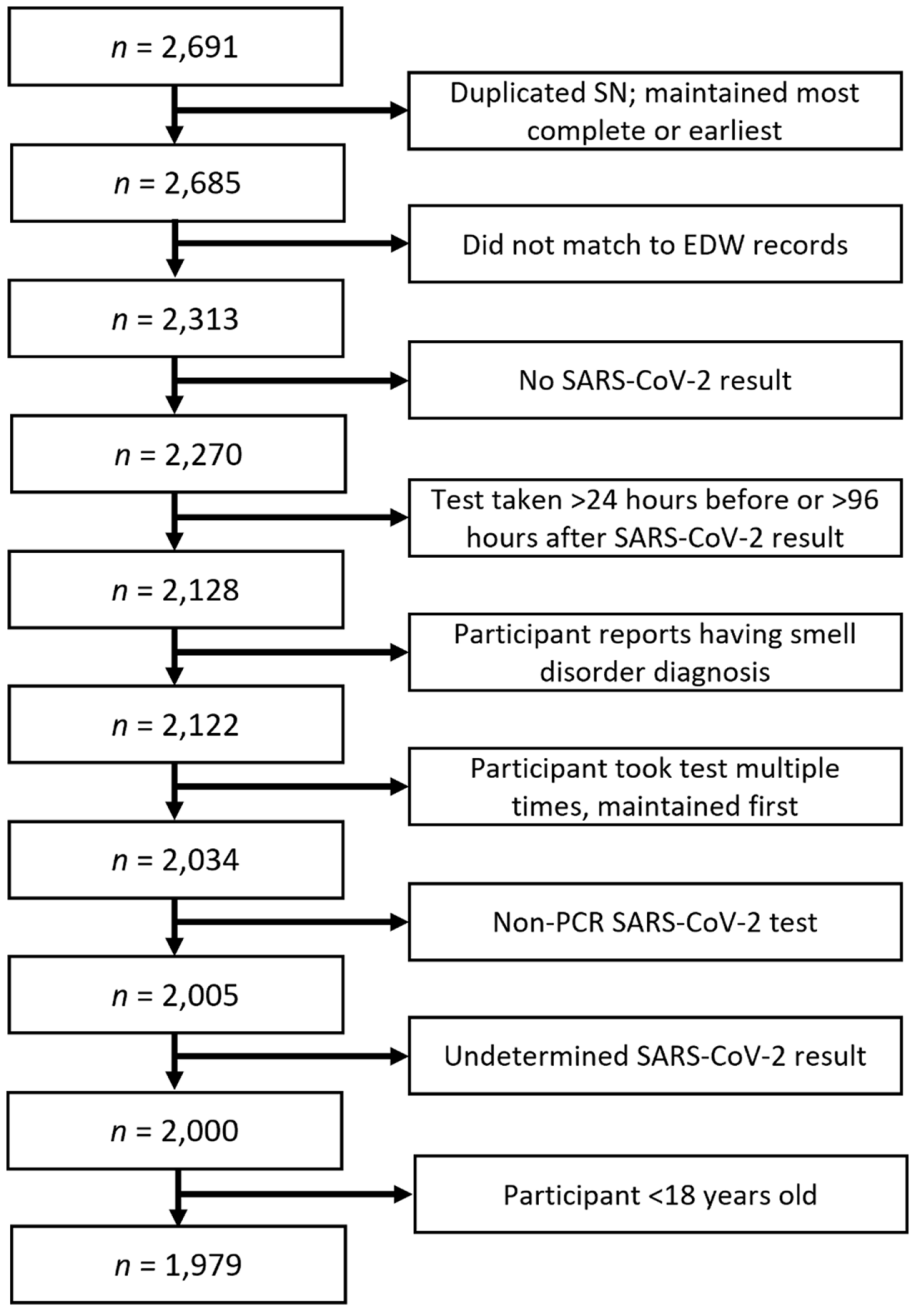
Exclusion criteria used to determine final sample size. EDW, Electronic data warehouse.

Participants were instructed to throw away their SCENTinel® test card after completing the survey, but due to the self-administered nature of the test, we were not able to ensure that this happened. When there were multiple entries for a single SCENTinel® test card (due to either one person taking the test multiple times in one sitting or different people, such as a family member, using the same test card), we only retained data from either the most complete or the earliest results from the same card; *n* = 6. Those who took the SCENTinel® test but did not have corresponding EDW record matches (*n* = 372) were excluded, leaving 2,313 SCENTinel® tests matched existing EDW records. Additionally, we excluded participants based on our preregistered criteria (41) to come to our final sample size. We removed all participants who (1) had no SARS-CoV-2 test results in their EDW (*n* = 43), (2) completed the SCENTinel® test either more than 24 h before or more than 96 h after the release of the SARS-CoV-2 result (*n* = 142), (3) had a self-reported preexisting smell disorder diagnosis (*n* = 6), (4) took multiple SCENTinel® tests over the 15-month study period (we only included their first completion of the test; *n* = 88), (5) took a non-PCR SARS-CoV-2 test (*n* = 29), (6) had an undetermined PCR test result (*n* = 5), or (7) were less than 18 years old (*n* = 21). The final sample size included 1,979 participants (Figure 1).

### SARS-CoV-2 variants

We defined periods of variant dominance as continuous, uninterrupted periods of time when a given SARS-CoV-2 variant consisted of 90% or more of cases each week. We gathered data on local variant prevalence via the Global Initiative on Sharing Avian Influenza Data, a publicly accessible repository for genetic sequences for influenza viruses such as coronaviruses (42). Available sequences were matched to the dates of data collection (April 5, 2021 to July 5, 2022), where Northwestern Memorial Hospital was the originating laboratory, and the location was listed as either Chicago, Cook County, or Lake County (39). We identified periods where one variant was dominant; periods when no COVID-19 variant was dominant in the community were labeled as Mixed.

### Data analysis

The analysis plan was preregistered (41); see Appendix S2 for all the analyses reported in the pre-registration. All statistical analyses were conducted in the R Environment for Statistical Computing (Version 4) (43).

To determine how well each SCENTinel® subtest, as well as the SCENTinel® overall score, predicted a positive SARS-CoV-2 infection, we calculated sensitivity (true positive rate), specificity (true negative rate), percent agreement, positive predictive value (likelihood of true positive if tested positive), negative predictive value (likelihood of true negative if tested negative), and kappa coefficients (a measure of agreement that corrects for chance agreement) between SCENTinel® subtests or overall score and SARS-CoV-2-positive test results. We calculated each agreement statistic across the full sample and for subsamples of tests taken during each wave of variant dominance (i.e., Mixed, Delta dominant, and Omicron dominant), since previous reports indicated different prevalence rates of smell loss across variants. Since SARS-CoV-2 positivity during the Delta-dominant period showed the best correspondence with SCENTinel® overall score and subtests (more people suffered from smell loss from this variant), we focused on this period for further analyses. We used Pearson correlations to investigate the correspondence between SCENTinel® overall or subtest scores and SARS-CoV-2 test results. Odor intensity was examined as both a dichotomous variable (≥21/≤20 pass/fail) and a continuous variable (0–100). Analyses for all variants can be found in Appendix S2.

We compared nested logistic regression models using likelihood ratio tests to assess whether the SCENTinel® test could predict SARS-CoV-2 infection above and beyond a count of self-reported symptoms, which included self-reported loss of taste or smell. First, we ran a logistic regression of the SARS-CoV-2 test result as the dependent variable and symptom count as the independent variable. Next, we ran two separate models with SARS-CoV-2 as the dependent variable and odor intensity (either dichotomous or continuous) and self-reported symptoms as the independent variables. We used McFadden’s pseudo-*R*^2^ and the Akaike information criterion (AIC) (44) to assess model fit. Due to the low positivity rate in our sample, the maximum likelihood estimates (MLEs) in the logistic regression may be biased. We therefore used the Firth method (45), also known as penalized MLE, to reduce small-sample bias in MLE by, in short, eliminating first-order bias (46). In the specific case of logistic regression, the Firth method will produce finite, consistent estimates of regression parameters when MLEs do not even exist, due to complete or quasi-complete separation.

## Results

### SCENTinel® shows high specificity and negative predictive value for SARS-CoV-2 infection

Of the 190 participants who failed the SCENTinel® overall score, 16 were C19+ (Table 3). Of the 1,764 participants who passed the SCENTinel® overall score, 1,657 were C19−. The sensitivity of the SCENTinel® overall score was low (13%), whereas the percent agreement (84.5%), specificity (90%), and negative predictive value (94%) were high (Table 4). Of the 222 subjects who failed the odor detection subtest (Table 3), 13 were C19+. Odor detection demonstrated poor sensitivity (10%) but good specificity (89%), agreement (83.7%), and negative predictive value (94%). Of the 548 participants who failed the odor identification subtest, 38 were C19+ (Table 3). Odor identification (first attempt) had a high negative predictive value (94%) and specificity (72%) but a lower sensitivity (31%). Odor identification and SARS-CoV-2 results agreed in 68.9% of cases. Of the 26 patients who failed the odor intensity subtest, 11 were C19+. Odor intensity thus showed high percentage agreement (93.0%), specificity (99%), and negative predictive value (94%) but low sensitivity (9%).

**Table 3:** SCENTinel® and SARS-CoV-2 cross-tabulations.

SCENTinel® test component	SARS-CoV-2 result (*n*)	
	Negative	Positive
Overall score		
Fail	174	16
Pass	1,657	107
Odor detection		
Fail	209	13
Pass	1,643	114
Odor identification		
Fail	510	38
Pass	1,325	86
Odor intensity		
Fail	16	11
Pass	1,830	116

**Table 4:** Agreement between SCENTinel® overall score and SARS-CoV-2 PCR test result by SARS-CoV-2 variant dominance.

	Overall	Mixed	Delta dominance	Omicron dominance
Time period	April 5, 2021 to July 5, 2022	April 5 2021 to July 24, 2021	July 25 2021 to November 18, 2021	December 10, 2021 to July 5, 2022
Kappa (*p* value)	0.71 (0.10)	0.63 (0.25)	0.89 (<0.001)	0.60 (0.51)
Percent agreement	85%	81%	93%	78%
Sensitivity	13%	21%	32%	3%
Specificity	90%	83%	97%	97%
Negative predictive value	94%	97%	97%	82%

### SCENTinel® overall score sensitivity was highest during SARS-CoV-2 Delta dominance

**Figure 2:**
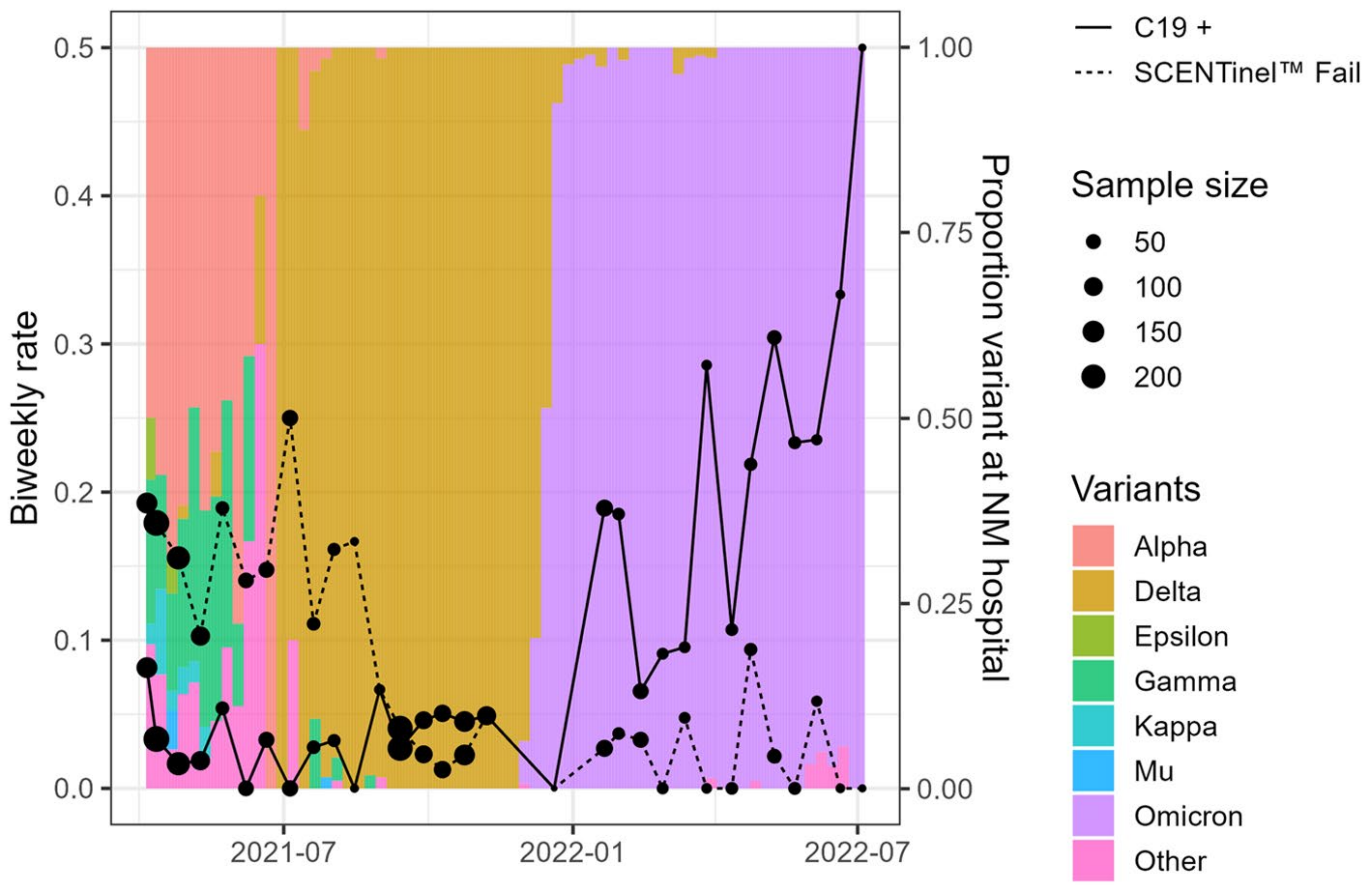
Overall SCENTinel® fail rate by SARS-CoV-2 positivity rate for each SARS-CoV-2 variant. The left axis represents our sample’s biweekly C19+ rate (solid line) and SCENTinel® failure rate (dashed line). The right axis and colored bars represent the proportion of each variant in Northwestern Medical hospital samples available via the Global Initiative on Sharing Avian Influenza Data repository.

Across the entire sample, 127 (6.4%) participants were C19+ during three distinct periods of COVID-19 variant dominance: 28 during the period of Mixed variants (April 5 to July 24, 2021), 28 during Delta dominance (July 25 to November 18, 2021), and 71 during Omicron dominance (December 10, 2021, to July 5, 2022). Sensitivity, percent agreement, specificity, and negative predictive value by SARS-CoV-2 variants differed, indicating that the SCENTinel® had different abilities to predict a positive SARS-CoV-2 test result depending on the SARS-CoV-2 variant that infected the individual (Table 4). Periods of COVID-19 variant dominance appear to be associated with heterogeneous effects in SCENTinel® test performance (Figure 2).

Specificity was highest for C19+ participants during Delta dominance (97%), driven primarily by results from the odor intensity subtest, which had a specificity of 99%. Odor intensity also showed the highest sensitivity among the subtests, at 36% (odor detection = 18%, odor identification = 32%). A failing SCENTinel® overall score showed greater agreement with C19+ status during Delta dominance (92.6%, Kappa = 0.89, *p* < 0.001) than during Omicron dominance (78.3% agreement, Kappa = 0.60, *p* = 0.51) or Mixed variants (80.8% agreement, Kappa = 0.63, *p* = 0.25; Table 4).

### Among SCENTinel® subtests, odor intensity was the best predictor of SARS-CoV-2 positivity during Delta dominance

**Figure 3:**
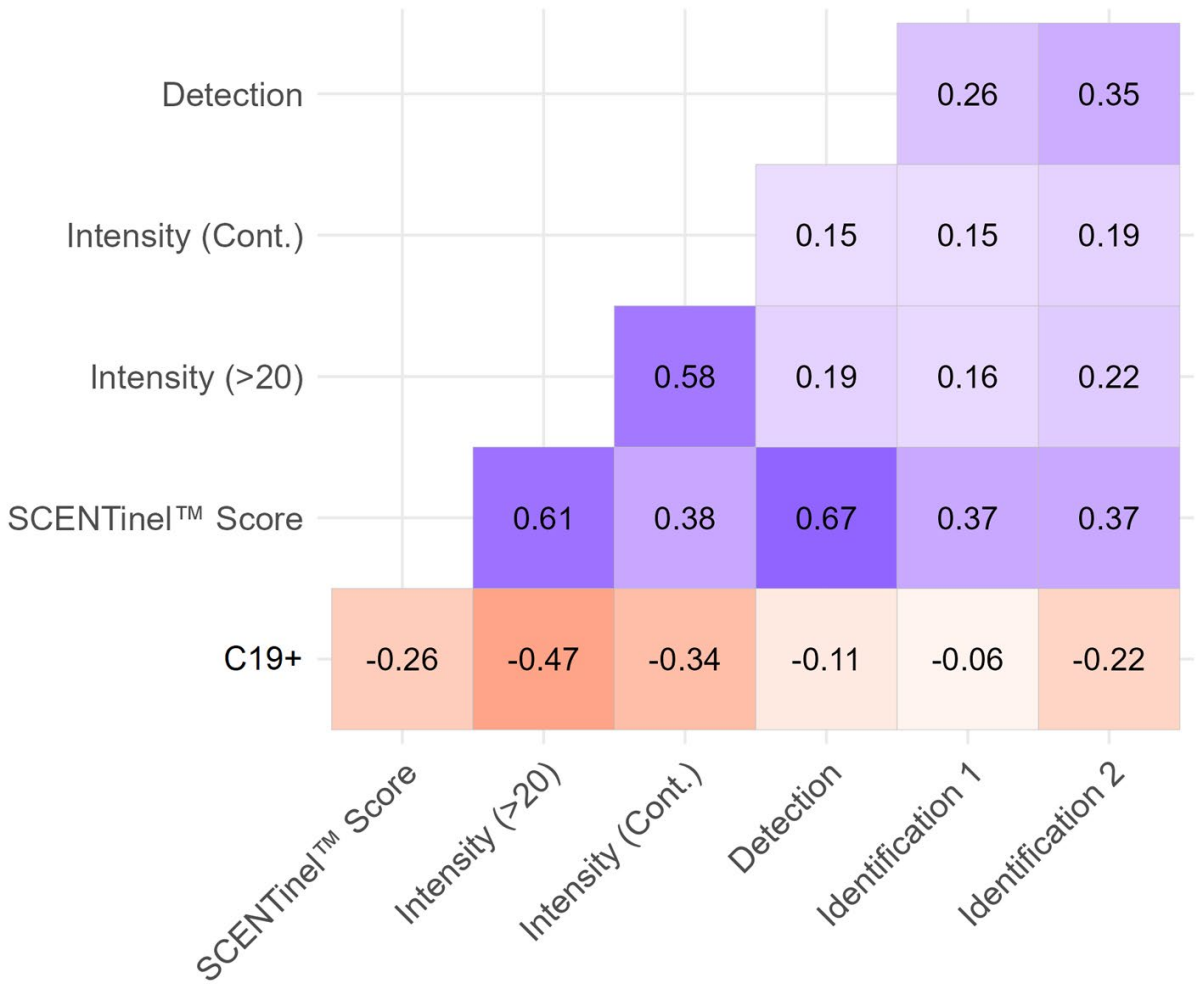
Heat map of correlations between C19+ and SCENTinel® test components for Delta-dominant period. All correlations greater than ±0.1 are significant at *p* < 0.05. All *N* = 693, except for the second identification attempt (Identification 2; *N* = 134).

SCENTinel® was most sensitive at predicting a positive PCR test result during Delta dominance, so we assessed correlations between SCENTinel® subtests and overall scores and SARS-CoV-2 test results during this period. As shown in Figure 3, odor detection had the highest correlation (*p* < 0.001), followed by dichotomized odor intensity (*p* < 0.001), continuous measure of odor intensity (*p* < 0.001), first odor identification (*p* < 0.001), and second odor identification (*p* < 0.001).

**Figure 4:**
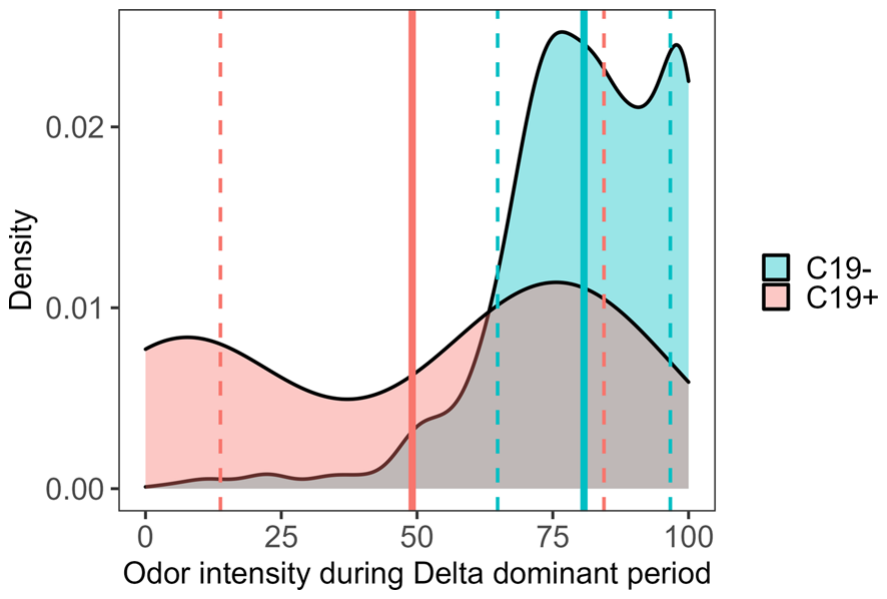
SCENTinel® odor intensity ratings between C19+ and C19− participants during the Delta-dominant period. SARS-CoV-2 positive: mean (solid line) = 49.11, SD (dashed lines) = 35.31; SARS-CoV-2 negative: mean = 80.74, SD = 15.90; Cohen’s *d* = 1.16.

A failing SCENTinel® overall score had a moderate negative correlation with C19+ results [*r*(693) = −0.26, *p* < 0.001], which implies that failing the SCENTinel® test is associated with SARS-CoV-2 positivity. Each SCENTinel® subtest also yielded interesting insights. For example, there was a strong negative correlation between C19 status and odor intensity, whether measured as a continuous variable [*r*(704) = −0.34, *p* < 0.001] or dichotomized [≤20, ≥21; *r*(704) = −0.47, *p* < 0.001; Figure 4]. Odor detection [*r*(705) = −0.11, *p* = 0.002] and first odor identification [*r*(695) = −0.06, *p* = 0.12] were weakly associated with C19 + .

### SCENTinel® odor intensity is a predictor of a positive SARS-CoV-2 test above and beyond self-reported symptoms

There were 576 participants (30%) who self-reported at least one symptom, and 110 C19+ participants (87%) self-reported at least one symptom. There was no correlation between self-reported smell loss and the SCENTinel® overall score (*r* = −0.01). Logistic regression models showed that of the SCENTinel® subtests, odor intensity (as a continuous variable) was a significant predictor of SARS-CoV-2 positivity, above and beyond self-reported symptoms, which includes self-reported smell loss (see Supplementary Table S3). A baseline model using symptoms alone to predict C19+, using Firth’s bias correction, had an AIC of 166.10 and a McFadden’s pseudo-*R*^2^ of 0.22 (Table 5). When odor intensity was added to this model as an independent variable, the model fit (AIC = 139.84, McFadden = 0.32) improved significantly [likelihood ratio test vs. 166.10: χ^2^(1) = 26.26, *p* < 0.001]. Adjusting for symptom count, a 10-point decrease in odor intensity resulted in a participant being 1.44 times more likely to be C19+ (95% CI = [1.21, 1.71]). A model including odor intensity as a dichotomous variable also improved model fit significantly over the baseline model [AIC = 150.69, McFadden pseudo-*R*-squared = 0.30, χ^2^(1) = 15.41, *p* < 0.001]. Adjusting for symptom count, failing the odor intensity subtest resulted in the participant being 17.94 times more likely to be C19+ (95% CI = [4.36, 75.82]).

**Table 5:** Model comparison.

Model		Model fit			Comparison to model 1	
		AIC	McFadden’s pseudo *R*^2^	Log-likelihood	χ^2^	*p* value
1	Symptoms (count)	166.1	0.22	−81.05		
2	Symptoms (count) + SCENTinel® odor intensity (continuous)	139.8	0.32	−67.92	26.26	< 0.001
3	Symptoms (count) + SCENTinel® odor intensity (pass/fail)	150.7	0.3	−73.35	15.41	< 0.001

“Symptoms (count)” refers to the number of all self-reported symptoms, including loss of taste and smell. For all models, the outcome variable was the SARS-CoV-2 test result. The sample size (*n* = 694) was restricted to the subset of participants that had complete data for Model 3 (i.e., using symptoms and dichotomized SCENTinel® odor intensity as independent variables).

## Discussion

While the prevalence of smell loss from active COVID-19 infection differed across SARS-CoV-2 variants, sudden smell loss is still a specific symptom of COVID-19 (7, 8, 17, 19, 20, 22, 47, 48). Directly measuring smell function can identify more cases of smell loss than can self-report alone (10). SCENTinel® is a rapid smell test that measures multiple olfactory functions and can discriminate among people with normosmia, anosmia, hyposmia, and parosmia (29, 34). The purpose of this study was to determine if SCENTinel® could screen for active SARS-CoV-2 infection.

We hypothesized that fewer C19+ vs. C19− participants would meet the accuracy criteria of the SCENTinel® odor detection and odor identification subtests, and overall score. These hypotheses were supported. However, a similar proportion of C19+ and C19− participants met the accuracy criteria of the SCENTinel® odor detection subtest. This could have been due to the high probability of guessing in this subtest, or that participants with C19+ could still smell, but it was diminished (i.e., hyposmia).

We also hypothesized that C19+ vs. C19− participants would rate the average odor intensity rating lower, which was supported. Prior studies using self-testing with odor items found in the home (e.g., spices, fragranced products) found odor intensity ratings were predictive of the prevalence of COVID-19 (47, 49), highlighting the usefulness of measures of odor intensity to identify smell loss. We also observed that the odor intensity subtest of SCENTinel® was most strongly correlated with active SARS-CoV-2 infection. Dichotomizing the odor intensity variable at 20/100 was particularly informative of active SARS-CoV-2 infection (8). Our findings supported the use of this cutoff and odor intensity ratings to determine active SARS-CoV-2 infection (49). The majority of commercially available smell tests only measure odor identification (28, 30), yet our results suggest a multifunctional smell test is needed not only to identify multiple olfactory disorders but also to detect a positive SARS-CoV-2 infection, particularly when variants with high rates of smell loss are dominant.

Given the sample size and duration of the study, the low number of positive SARS-CoV-2 cases was surprising. One possible explanation for the low number of positive cases is that individuals who tested positive felt so unwell that they opted to not participate in this study. Another possible explanation for the low number of positive cases lies in the reason participants were tested for SARS-CoV-2. Twenty-seven percent of participants were tested in order to undergo scheduled procedures (Supplementary Table S1), not necessarily because of COVID-19 symptoms, and may have been more cautious leading up to the procedure to avoid getting SARS-CoV-2.

Although SCENTinel® was more sensitive at screening for SARS-CoV-2 Delta-variant positivity than for the Omicron positivity, the sensitivity rate was still low across this 15-month study. There are several reasons why this may be the case. First, we do not know at what point in their illness participants were tested for SARS-CoV-2 or the timeline for appearance of their symptoms. Varied reports state the appearance of smell loss as the first and only symptom, or a delayed appearance of smell loss compared to other symptoms (7, 50–58). Participants who tested positive for SARS-CoV-2 and then lost their sense of smell several days later would not have failed SCENTinel® at the point-of-contact test. It would have been useful to know a person’s baseline smell function, to see how much and when they deviated from it, which could improve the sensitivity of the test. Unfortunately, to date, smell function is not routinely assessed, and this data is only sporadically included in medical records. Second, while PCR is widely regarded as the gold standard, false-negative rates of reverse-transcriptase PCR are high (59), partly due to the time of sampling after appearance of symptoms, or differences in viral load in different body regions (60, 61). Third, human error in either test administration or RNA extraction may contribute to the relatively high rate of false negatives observed in our community sample. Nonetheless, the specificity and negative predictive value were high, suggesting that SCENTinel® can be a useful tool to determine who does not have SARS-CoV-2. There are many such examples of tests with high specificity but low sensitivity currently developed and used in clinical-level practice; these include using Ankle-Brachial Index (ABI), an anthropometric calculation, as a potential marker for peripheral arterial disease and potentially as an indicator of cardiovascular risk (62), Pap smears for screening for cervical cancer (63), and certain blood tests to detect blood-based breast cancer (64). Thus, screening tests with low sensitivity but high specificity are still widely useful for clinical care (62).

Lastly, we hypothesized that SCENTinel® test alone or in conjunction with other available data would correctly predict C19+ vs. C19− status, which was supported. When adjusting for self-reported symptom count (including self-reported taste and smell loss), failing the odor intensity subtest of SCENTinel® resulted in the participant being almost 18 times more likely to be C19+ when Delta was the dominant variant. Even though odor intensity is a subjective measure, it provides a few advantages compared to the more frequently used self-report of smell function. First, it capitalizes on the exposure to a standardized odor, which has consistently been reported having an intensity of 80/100 among people with a normal sense of smell (29, 34). Second, by asking participants to directly smell an odor, it offers them the opportunity to test whether a change in smell function has occurred. For participants to self-report smell loss, they must be aware of the change and we know that many people do not notice this, particularly when the loss is subtle (i.e., in hyposmia) or gradual (i.e., with aging). Self-reported symptoms have often been used as screening tools throughout the pandemic to determine if someone might be infected with SARS-CoV-2 (6). Our results indicate that using self-reported symptoms is not sufficient, and asking how intense a standard odor is, even though it is a subjective metric, is a useful tool to screen for olfactory changes, including in the COVID-19 pandemic.

Although SCENTinel® had a low sensitivity for predicting SARS-CoV-2 on an individual basis, it was still able to track the SARS-CoV-2 positivity rate in the population during Mixed and Delta variant dominance, suggesting that olfactory testing can be a useful metric for surveillance when smell loss is associated with a viral infection, such as COVID-19. These findings prompt increased focus on the value of population-wide screening of olfactory function for current and future disease. Problems were encountered in developing easily administered SARS-CoV-2 tests early in the pandemic, which delayed the ability to determine if and for how long someone was contagious and prevented health agencies from accurately assessing the prevalence of COVID-19 for months (65). Smell loss has been a symptom in several viral illnesses, particularly in coronavirus-related diseases (66) and is likely to be a symptom in a future viral pandemic (67, 68). Because it is self-administered, rapid and inexpensive, SCENTinel® can easily be deployed in the population to track smell function.

## Conclusion

Although SCENTinel® had sensitivity of only 30% for identifying a positive SARS-CoV-2 infection, it was predictive of SARS-CoV-2 positivity above and beyond self-reported symptoms alone, and had a high negative predictive value, indicating that those who passed SCENTinel® likely did not have a SARS-CoV-2 infection. Odor intensity in particular could predict positive SARS-CoV-2, which many commercially available smell tests do not measure. Regularly implementing smell testing with SCENTinel® in routine clinical care can establish baseline values of smell function, which may be helpful in future viral pandemics to determine when someone is deviating from baseline and possibly have a viral infection, and can provide population-wide data to signal early signs of widespread viral infections.

## Data availability statement

The original contributions presented in the study are publicly available. This data can be found here: The Open Science Framework (OSF), https://osf.io/, DOI: 10.17605/OSF.IO/5R9JB.

## Ethics statement

The studies involving humans were approved by the University of Pennsylvania Institutional Review Board as the IRB of record (protocol no. 844425). The studies were conducted in accordance with the local legislation and institutional requirements. The participants provided their written informed consent to participate in this study.

## Author contributions

SH: Data curation, Investigation, Project administration, Supervision, Validation, Writing – original draft, Writing – review & editing. AZ: Data curation, Formal analysis, Investigation, Validation, Visualization, Writing – original draft, Writing – review & editing. EH: Conceptualization, Funding acquisition, Project administration, Supervision, Writing – original draft, Writing – review & editing. MK: Conceptualization, Formal analysis, Funding acquisition, Methodology, Supervision, Writing – review & editing. EA-D: Investigation, Writing – review & editing. CA: Investigation, Resources, Writing – review & editing. GS: Conceptualization, Funding acquisition, Writing – review & editing. RG: Conceptualization, Funding acquisition, Writing – review & editing. DR: Conceptualization, Funding acquisition, Writing – review & editing. BS: Conceptualization, Funding acquisition, Methodology, Project administration, Supervision, Writing – review & editing. VP: Conceptualization, Funding acquisition, Methodology, Project administration, Supervision, Writing – original draft, Writing – review & editing. PD: Conceptualization, Funding acquisition, Methodology, Project administration, Supervision, Writing – review & editing.

## Supplementary material

Supplementary Figure S1 The SCENTinel^®^ test card (version 2.0), with Lift’nSmell technology (Scentisphere, Carmel, NY).

Supplementary Figure S2 SCENTinel^®^ Test Survey example.

Supplementary Table S1 Population of participants tested.

Supplementary Table S2 Symptom endorsement by SARS-CoV-2 positivity status and variant dominance. All symptoms were significantly different (*p* ≤ 0.05) between those who tested negative and positive for SARS-CoV-2, except for cough, congestion/runny nose, and diarrhea in Mixed variant dominance; cough in Delta dominance; and sore throat and nausea/vomiting in Omicron dominance.

Supplementary Table S3 Each test contained only one of these odors. Odors are from ^1^Givaudan, Cincinnati, OH or ^2^Robertet, Mount Olive, NJ.

Supplementary Appendix S1 SARS-CoV-2 PCR test results and SCENTinel^®^ test result matching.

Supplementary Appendix S2 All pre-registered analyses.
