# Supplementary material for: Using SCENTinel® to predict SARS-CoV-2 infection: insights from a community sample during dominance of Delta and Omicron variants
Source: Front Public Health. 2024 Apr 10;12:1322797. doi: 10.3389/fpubh.2024.1322797 (PMC11041634; doi:10.3389/fpubh.2024.1322797)
Supplement: Supplementary Figure S1 — The SCENTinel® test card (version 2.0), with Lift’nSmell technology (Scentisphere, Carmel, NY). [file Data_Sheet_1.PDF]

S3 Table.

	Total		Mixed Dominance		Delta dominance		Omicron dominance	
	Negative	Positive	Negative	Positive	Negative	Positive	Negative	Positive
N	1852	127	858	28	678	28	316	71
Fever or chills	213 (11.5)	63 (49.6)	77 (9.0)	9 (32.1)	90 (13.3)	14 (50.0)	46 (14.6)	40 (56.3)
Cough	126 (6.8)	28 (22.0)	50 (5.8)	3 (10.7)	48 (7.1)	4 (14.3)	28 (8.9)	21 (29.6)
Shortness of breath	145 (7.8)	65 (51.2)	57 (6.6)	10 (35.7)	60 (8.8)	13 (46.4)	28 (8.9)	42 (59.2)
Fatigue	254 (13.7)	68 (53.5)	81 (9.4)	12 (42.9)	114 (16.8)	11 (39.3)	59 (18.7)	45 (63.4)
Muscle or body aches	96 (5.2)	43 (33.9)	24 (2.8)	11 (39.3)	51 (7.5)	12 (42.9)	21 (6.6)	20 (28.2)
Headache	36 (1.9)	20 (15.7)	9 (1.0)	5 (17.9)	20 (2.9)	6 (21.4)	7 (2.2)	9 (12.7)
New loss of taste/smell	128 (6.9)	42 (33.1)	44 (5.1)	10 (35.7)	62 (9.1)	10 (35.7)	22 (7.0)	22 (31.0)
Sore throat	94 (5.1)	16 (12.6)	29 (3.4)	6 (21.4)	47 (6.9)	6 (21.4)	18 (5.7)	4 (5.6)
Congestion/runny nose	32 (1.7)	15 (11.8)	13 (1.5)	2 (7.1)	15 (2.2)	4 (14.3)	4 (1.3)	9 (12.7)
Nausea or vomiting	28 (1.5)	24 (18.9)	11 (1.3)	4 (14.3)	13 (1.9)	16 (57.1)	4 (1.3)	4 (5.6)
Diarrhea	32 (1.7)	12 (9.4)	14 (1.6)	2 (7.1)	13 (1.9)	4 (14.3)	5 (1.6)	6 (8.5)
None	1386 (74.8)	17 (13.4)	685 (79.8)	8 (28.6)	481 (70.9)	2 (7.1)	220 (69.6)	7 (9.9)
