# Supplementary material for: Using SCENTinel® to predict SARS-CoV-2 infection: insights from a community sample during dominance of Delta and Omicron variants
Source: Front Public Health. 2024 Apr 10;12:1322797. doi: 10.3389/fpubh.2024.1322797 (PMC11041634; doi:10.3389/fpubh.2024.1322797)
Supplement: Supplementary Figure S2 — SCENTinel® Test Survey example. [file Data_Sheet_2.PDF]

S2 Table.

Odor	SCENTinel™ Test Version	Components
Flower[1]	1.1 and 2.0	2-Phenylethanol [CAS 60-12-8]<br>Benzoic acid<br>Phenyl methyl ester [CAS 120-51-4]<br>Linalool [CAS 78-70-6]<br>Geraniol [CAS 106-24-1]<br>Citronellol [CAS 106-22-9]<br>Nerol [CAS 106-25-2]<br>Geranyl acetate [CAS 105-87-3]<br>Rose oxide L [CAS 16409-43-1]<br>Methyl 2-nonynoate [CAS 111-80-8]
Coffee[1]	1.1	2-Methylpropanal [CAS 78-84-2]<br>Ethyl vanillin [CAS 121-32-4]
Coffee[2]	2.0	Vanillin [CAS 121-33-5]<br>Benzaldehyde [CAS 100-52-7]<br>Benzyl benzoate [CAS 120-51-4]<br>gamma-Hexalactone [CAS 695-06-7]<br>Ethyl vanillin [CAS 121-32-4]<br>Ethyl maltol [CAS 4940-11-8]<br>3-Methylbutyraldehyde [CAS 590-86-3]<br>Acetyl propionyl [CAS 600-14-6]
Bubblegum[1]	1.1 and 2.0	Phenyl methyl ester [CAS 120-51-4]<br>Cyclohexene [CAS 5989-27-5]<br>Vanillin [CAS 121-33-5]<br>Ethyl propionate [CAS 105-37-3]<br>Ethyl butyrate [CAS 105-54-4]<br>beta-Pinene [CAS 127-92-2]<br>Isoamyl acetate [CAS 123-92-2]<br>Myrcene [CAS 123-35-3]<br>Cinnamic aldehyde [CAS 104-55-2]
Caramel popcorn[1]	1.1	Acetoin dimer [CAS 513-86-0]<br>Ethyl vanillin [CAS 121-32-4]<br>Ethyl maltol [CAS 4940-11-8]<br>Vanillin [CAS 121-33-5]<br>Piperonal [CAS 120-57-0]
Orange[2]	2.0	Limonene [CAS 5989-27-5]<br>Methyl dihydrojasmonate [CAS 24851-98-7]<br>Ethyl methylphenylglycidate [CAS 77-83-8]<br>beta-Ionone [CAS 14901-07-6]<br>2-Butanone, 4-(4-hydroxyphenyl)- [CAS 5471-51-2]<br>Citral [CAS 5392-40-5]<br>alpha-Methylbenzyl acetate [CAS 93-92-5]<br>Ethyl maltol [CAS 4940-11-8]<br>Allyl heptanoate [CAS 142-19-8]<br>Linalool [CAS 78-70-6]<br>Hydroxycitronellol [CAS 107-74-7]<br>Decanal [CAS 112-31-2]<br>1-Methyl-4-(4-methyl-3-pentenyl)cyclohex-3-ene-1-carbaldehyde [CAS 52475-86-2]<br>Isohexenyl cyclohexenyl carboxaldehyde [CAS 37677-14-8]<br>beta-Myrcene [CAS 123-35-3]<br>Methoxy dicyclopentadiene carboxaldehyde [CAS 86803-90-9]<br>beta-Pinene [CAS 127-91-3]<br>Citronellol [CAS 106-22-9]<br>alpha-Pinene [CAS 80-56-8]<br>1-(2,6,6-Trimethylcyclohexa-1,3-dienyl)-2-buten-1-one [CAS 23696-85-7]

Strawberry[2]	2.0	Benzyl benzoate [CAS 120-51-4]<br>Ethyl 2-methyl-1,3-dioxolane-2-acetate [CAS 6413-10-1]<br>Ethyl methylphenylglycidate [CAS 77-83-8]<br>Ethylene brassylate [CAS 105-95-3]<br>gamma-Undecalactone [CAS 104-67-6]<br>Benzyl isobutyrate [CAS 103-28-6]<br>Limonene [CAS 5989-27-5]<br>Benzyl acetate [CAS 140-11-4]<br>Ethyl maltol [CAS 4940-11-8]<br>Ethyl butyrate [CAS 105-54-4]<br>beta-Pinene [CAS 127-91-3]<br>p-Mentha-1,4-diene [CAS 99-85-4]<br>Methyl cinnamate [CAS 103-26-4]<br>Citral [CAS 5392-40-5]<br>alpha-Pinene [CAS 80-56-8]<br>Benzyl salicylate [CAS 118-58-1]
Banana[2]	2.0	Methyl dihydrojasmonate [CAS 24851-98-7]<br>Isoamyl acetate [CAS 123-92-2]<br>Ethyl vanillin [CAS 121-32-4]<br>Benzyl benzoate [CAS 120-51-4]<br>Vanillin [CAS 121-33-5]<br>p-Methoxybenzaldehyde [CAS 123-11-5]<br>Limonene [CAS 5989-27-5]<br>Piperonal [CAS 120-57-0]<br>Butylated hydroxytoluene [CAS 128-37-0]<br>Ethyl butyrate [CAS 105-54-4]<br>Coumarin [CAS 91-64-5]<br>Benzyl acetate [CAS 140-11-4]<br>Allyl hexanoate [CAS 123-68-2]<br>2-Butanone, 4-(4-hydroxyphenyl)- [CAS 5471-51-2]<br>Ethyl maltol [CAS 4940-11-8]<br>Ethyl methylphenylglycidate [CAS 77-83-8]
Woody[2]	2.0	Benzyl benzoate [CAS 120-51-4]<br>Octahydronaphthalen-2-yl)ethanone [CAS 54464-57-2]<br>Hexyl cinnamal [CAS 101-86-0]<br>Cedrene [CAS 11028-42-5]<br>Acetyl cedrane [CAS 32388-55-9]<br>Isobornyl acetate [CAS 125-12-2]<br>Cedrol [CAS 77-53-2]<br>Limonene [CAS 5989-27-5]<br>Linalyl acetate [CAS 115-95-7]<br>1-(2-tert-Butyl cyclohexyloxy)-2-butanol [CAS 139504-68-0]<br>Linalool [CAS 78-70-6]<br>Dimethylcyclohex-3-ene-1-carbaldehyde [CAS 68737-61-1]<br>1,2,3,3a,4,5,6,8a-Octahydro-4,8-dimethyl-2-(1-methylethylidene)-6-azulenol [CAS 89-88-3]<br>Methyl atrarate [CAS 4707-47-5]<br>Eucalyptol [CAS 470-82-6]<br>Eugenol [CAS 97-53-0]<br>Benzyl cinnamate [CAS 103-41-3]<br>alpha-Pinene [CAS 80-56-8]<br>Camphene [CAS 79-92-5]<br>beta-Caryophyllene [CAS 87-44-5]<br>Longifolene [CAS 475-20-7]

Coconut[2]	2.0	Benzyl benzoate [CAS 120-51-4]<br>gamma-Nonalactone [104-61-0]<br>Methyl dihydrojasmonate [CAS 24851-98-7]<br>Ethyl vanillin [CAS 121-32-4]<br>gamma-Octalactone [CAS 104-50-7]<br>Ethylene brassylate [CAS 105-95-3]<br>Octahydronaphthalen-2-yl)ethanone [CAS 54464-57-2]<br>2-Methyl-3-p-isopropylphenyl)propionaldehyde [CAS 103-95-7]<br>2-Isobutyl-4-methyltetrahydro-2H-pyran-4-ol [CAS 63500-71-0]<br>Vanillin [CAS 121-33-5]<br>Coumarin [CAS 91-64-5]<br>Ethyl maltol [CAS 4940-11-8]<br>Linalool [CAS 78-70-6]<br>Piperonal [CAS 120-57-0]<br>gamma-Undecalactone [CAS 104-67-6]<br>Benzyl salicylate [118-58-1]
Lemon[2]	2.0	Benzyl benzoate [CAS 120-51-4]<br>Citral [CAS 5392-40-5]<br>Methyl dihydrojasmonate [CAS 24851-98-7]<br>Limonene [CAS 5989-27-5]<br>Hexamethylindanopyran [CAS 1222-05-5]<br>Ethyl maltol [CAS 4940-11-8]<br>beta-Pinene [CAS 127-91-3]<br>3,7-Demethyl-2,6-nonadienenitrile [CAS 61792-11-8]<br>p-Mentha-1,4-diene [CAS 99-85-4]<br>Ethylene brassylate [CAS 105-95-3]<br>beta-Myrcene [CAS 123-35-3]<br>alpha-Pinene [CAS 80-56-8]<br>2,6-Demethyl-5-heptenal [CAS 106-72-9]<br>Benzyl salicylate [CAS 118-58-1]
