# Supplementary figures and images for: Using SCENTinel® to predict SARS-CoV-2 infection: insights from a community sample during dominance of Delta and Omicron variants
Source: Front Public Health. 2024 Apr 10;12:1322797. doi: 10.3389/fpubh.2024.1322797 (PMC11041634; doi:10.3389/fpubh.2024.1322797)

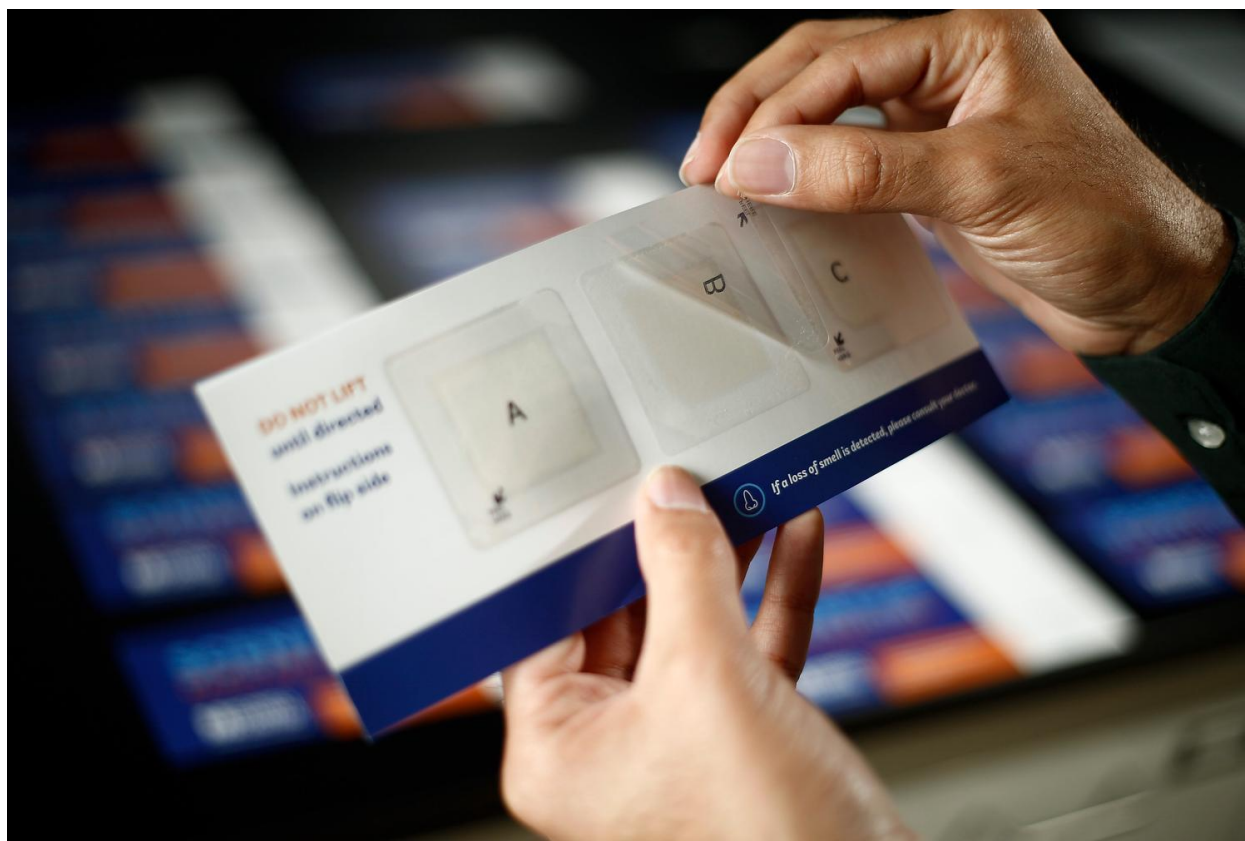

S1 Fig.

Supplement: Supplementary Table S1 — Population of participants tested. [file Data_Sheet_3.PDF]
