# Supplementary material for: Using SCENTinel® to predict SARS-CoV-2 infection: insights from a community sample during dominance of Delta and Omicron variants
Source: Front Public Health. 2024 Apr 10;12:1322797. doi: 10.3389/fpubh.2024.1322797 (PMC11041634; doi:10.3389/fpubh.2024.1322797)
Supplement: Supplementary Table S2 — Symptom endorsement by SARS-CoV-2 positivity status and variant dominance. All symptoms were significantly different (p ≤ 0.05) between those who tested negative and positive for SARS-CoV-2, except for cough, congestion/runny nose, and diarrhea in Mixed variant dominance; cough in Delta dominance; and sore throat and nausea/vomiting in Omicron dominance. [file Data_Sheet_4.PDF]

S1 Appendix.

Participants self-reported first name, middle name, last name, and date of birth (DOB). First name, last name, and DOB were used to match to existing health records. If the attempt to match participant records was unsuccessful, we cleaned records using the following rules (in order) to increase chances of matching.

1. If there was a date in a name field (first or last name), the date was moved to the DOB field.
2. We removed any numbers from name fields.
3. If last name was 1 character or less and the middle name was more than 1 character, the middle name was moved to the last name field.
4. We removed all suffixes (e.g., iv, jr, sr).
5. We edited DOB four-digit years using the following rules, in order:
    1. 21[3-9][0-9] → 19[3-9][0-9]
    2. 2[1-9]?? → 1[1-9]??
    3. 1[0-8]?? → 19??
    4. 91?? → 19??
6. We then created multiple versions of the record using the following rules and submitted all versions for attempted matching.

    a. If the 2-digit month and the 2-digit day could be switched to make a valid date, we created a version with the month and day switched.

    b. If the name fields contained special characters (e.g., á), we created a version with no special characters (e.g., a).

    c. If the last name was hyphenated, we created one version where the last name was the name before the hyphen only and another version where last name was the name after the hyphen only.

    d. If there were apostrophes in the name fields (e.g., o'donnell), we created a version with apostrophes removed (e.g., odonnell).

    e. We created versions of first name variations based an open source nicknames database, https://github.com/carltonnorthern/nicknames (retrieved November 19th, 2021)
