# Supplementary material for: Using SCENTinel® to predict SARS-CoV-2 infection: insights from a community sample during dominance of Delta and Omicron variants
Source: Front Public Health. 2024 Apr 10;12:1322797. doi: 10.3389/fpubh.2024.1322797 (PMC11041634; doi:10.3389/fpubh.2024.1322797)
Supplement: Supplementary Table S3 — Each test contained only one of these odors. Odors are from 1Givaudan, Cincinnati, OH or 2Robertet, Mount Olive, NJ. [file Data_Sheet_5.PDF]

S1 Table.

Population	n (%)
Outpatient community	619 (31.3)
Pre-testing scheduled procedures	534 (27.0)
Northwestern Medicine employees	82 (4.1)
Immunocompromised	38 (1.9)
Unknown	706 (35.7)
