# Supplementary material for: Using SCENTinel® to predict SARS-CoV-2 infection: insights from a community sample during dominance of Delta and Omicron variants
Source: Front Public Health. 2024 Apr 10;12:1322797. doi: 10.3389/fpubh.2024.1322797 (PMC11041634; doi:10.3389/fpubh.2024.1322797)
Supplement: Supplementary Appendix S1 — SARS-CoV-2 PCR test results and SCENTinel® test result matching. [file Data_Sheet_6.PDF]

SCENTinel & SARS-CoV-2 Infection

04 May, 2023

Overall sample

SARS-CoV-2 results

Table 1: SCV-2 infection

	n
NEGATIVE	1852
POSITIVE	127

Symptoms

Table 2: Symptoms

	NEGATIVE	POSITIVE	p	test
n	1852	127		
Fever or chills = 1 (%)	213 (11.5)	63 (49.6)	<0.001	
Cough = 1 (%)	126 (6.8)	28 (22.0)	<0.001	
Shortness of breath = 1 (%)	145 (7.8)	65 (51.2)	<0.001	
Fatigue = 1 (%)	254 (13.7)	68 (53.5)	<0.001	
Muscle or body aches = 1 (%)	96 (5.2)	43 (33.9)	<0.001	
Headache = 1 (%)	36 (1.9)	20 (15.7)	<0.001	
New loss of taste/smell = 1 (%)	128 (6.9)	42 (33.1)	<0.001	
Sore throat = 1 (%)	94 (5.1)	16 (12.6)	0.001	
Congestion/runny nose = 1 (%)	32 (1.7)	15 (11.8)	<0.001	
Nausea or vomiting = 1 (%)	28 (1.5)	24 (18.9)	<0.001	
Diarrhea = 1 (%)	32 (1.7)	12 (9.4)	<0.001	
None = 1 (%)	1386 (74.8)	17 (13.4)	<0.001	

Correlations

	SARS-CoV-2+	SCENTinel Score	Intensity (>20)	Intensity (Cont.)	Detection	Identification 1	Identification 2
SARS-CoV-2+	1.00	-0.03	-0.16	-0.14	0.01	-0.02	-0.02
SCENTinel Score	-0.03	1.00	0.34	0.16	0.84	0.51	0.39

	SARS-CoV-2+	SCENTinel Score	Intensity (>20)	Intensity (Cont.)	Detection	Identification 1	Identification 2
Intensity (>20)	-0.16	0.34	1.00	0.48	0.08	0.13	0.08
Intensity (Cont.)	-0.14	0.16	0.48	1.00	0.06	0.15	0.03
Detection	0.01	0.84	0.08	0.06	1.00	0.41	0.38
Identification 1	-0.02	0.51	0.13	0.15	0.41	1.00	NA
Identification 2	-0.02	0.39	0.08	0.03	0.38	NA	1.00

Agreement statistics

Odor detection

Table 4: Agreement between COVID Result and Odor detection

	NEGATIVE	POSITIVE
Fail	209	13
Pass	1643	114

Table 5: Prevalance Adjusted Kappa (95% CI)

Estimate	Lower	Upper
0.674	0.64	0.706

Table 6: Prevalance Adjusted Kappa (Test statistic)

z test statistic	p-value
-0.36233	0.64145

Percent agreement: 83.68%
Sensitivity: 0.1
Specificity: 0.89
Negative Predictive Value: 0.94

Odor intensity

Table 7: Agreement between COVID Result and Odor intensity

	NEGATIVE	POSITIVE
	6	0
Fail	16	11

	NEGATIVE	POSITIVE
Pass	1830	116

Table 8: Prevalance Adjusted Kappa (95% CI)

Estimate	Lower	Upper
0.866	0.842	0.887

Table 9: Prevalance Adjusted Kappa (Test statistic)

z test statistic	p-value
7.31351	0

Percent agreement: 93.03%
Sensitivity: 0.09
Specificity: 0.99
Negative Predictive Value: 0.94

Odor identification

First try at identification only.

Table 10: Agreement between COVID Result and Odor identification

	NEGATIVE	POSITIVE
	17	3
Fail	510	38
Pass	1325	86

Table 11: Prevalance Adjusted Kappa (95% CI)

Estimate	Lower	Upper
0.392	0.35	0.432

Table 12: Prevalance Adjusted Kappa (Test statistic)

z test statistic	p-value
0.68482	0.24673

Percent agreement: 68.87%
Sensitivity: 0.3
Specificity: 0.72
Negative Predictive Value: 0.94

SCENTinel Score

Table 13: Agreement between COVID Result and SCENTinel score

	NEGATIVE	POSITIVE
	21	4
Fail	174	16
Pass	1657	107

Table 14: Prevalance Adjusted Kappa (95% CI)

Estimate	Lower	Upper
0.712	0.68	0.743

Table 15: Prevalance Adjusted Kappa (Test statistic)

z test statistic	p-value
1.2701	0.10203

Percent agreement: 84.54%
Sensitivity: 0.13
Specificity: 0.89
Negative Predictive Value: 0.94

Logistic regression

```
## logistf(formula = covid_factor ~ odor_discrim_correct + odor_intensity_correct +
##     odor_ident_correct_1, data = data2, na.action = na.exclude,
##     family = binomial)
##
## Model fitted by Penalized ML
## Coefficients:
##                              coef  se(coef) lower 0.95  upper 0.95      Chisq
## (Intercept)            -0.8518607 0.4755642 -1.8370238  0.05031282  3.4157228
## odor_discrim_correct    0.3164564 0.3391430 -0.3193746  1.02468702  0.9080164
## odor_intensity_correct -2.1012530 0.4464330 -2.9570027 -1.18220485 17.0105537
## odor_ident_correct_1   -0.1006366 0.2240592 -0.5298753  0.35308882  0.1981308
##                                  p method
## (Intercept)            6.457813e-02      2
## odor_discrim_correct   3.406413e-01      2
## odor_intensity_correct 3.717263e-05      2
## odor_ident_correct_1   6.562338e-01      2
##
## Method: 1-Wald, 2-Profile penalized log-likelihood, 3-None
##
## Likelihood ratio test=18.25353 on 3 df, p=0.0003899401, n=1958
## Wald test = 829.4033 on 3 df, p = 0
```

```
## [1] "AIC: 899.55"
```

```
## [1] "McF pseudo-R: 0.02"
```

```
## logistf(formula = covid_factor ~ odor_discrim_correct + odor_intensity +
##     odor_ident_correct_1, data = data2, na.action = na.exclude,
##     family = binomial)
##
## Model fitted by Penalized ML
## Coefficients:
##                             coef    se(coef)  lower 0.95  upper 0.95
## (Intercept)          -1.11945124 0.414300596 -1.97241669 -0.33505994
## odor_discrim_correct  0.26005487 0.334433689 -0.36727410  0.95880406
## odor_intensity       -0.02327490 0.004533557 -0.03209962 -0.01419838
## odor_ident_correct_1 -0.04208122 0.222115209 -0.46923565  0.40670899
##                            Chisq            p method
## (Intercept)           8.04586541 4.560763e-03      2
## odor_discrim_correct  0.62501432 4.291900e-01      2
## odor_intensity       23.68653786 1.133728e-06      2
## odor_ident_correct_1  0.03545825 8.506387e-01      2
##
## Method: 1-Wald, 2-Profile penalized log-likelihood, 3-None
##
## Likelihood ratio test=24.88993 on 3 df, p=1.628072e-05, n=1958
## Wald test = 808.8612 on 3 df, p = 0
```

```
## [1] "AIC: 882.82"
```

```
## [1] "McF pseudo-R: 0.0275"
```

Mixed variants sample

SARS-CoV-2 results

Table 16: SCV-2 infection

	n
NEGATIVE	858
POSITIVE	28

Symptoms

Table 17: Symptoms

	NEGATIVE	POSITIVE	p	test
n	858	28		
Fever or chills = 1 (%)	77 (9.0)	9 (32.1)	<0.001	
Cough = 1 (%)	50 (5.8)	3 (10.7)	0.504	

	NEGATIVE	POSITIVE	p	test
Shortness of breath = 1 (%)	57 (6.6)	10 (35.7)	<0.001	
Fatigue = 1 (%)	81 (9.4)	12 (42.9)	<0.001	
Muscle or body aches = 1 (%)	24 (2.8)	11 (39.3)	<0.001	
Headache = 1 (%)	9 (1.0)	5 (17.9)	<0.001	
New loss of taste/smell = 1 (%)	44 (5.1)	10 (35.7)	<0.001	
Sore throat = 1 (%)	29 (3.4)	6 (21.4)	<0.001	
Congestion/runny nose = 1 (%)	13 (1.5)	2 (7.1)	0.127	
Nausea or vomiting = 1 (%)	11 (1.3)	4 (14.3)	<0.001	
Diarrhea = 1 (%)	14 (1.6)	2 (7.1)	0.152	
None = 1 (%)	685 (79.8)	8 (28.6)	<0.001	

Correlations

	SARS-CoV-2+	SCENTinel Score	Intensity (>20)	Intensity (Cont.)	Detection	Identification 1	Identification 2
SARS-CoV-2+	1.00	-0.02	-0.04	-0.07	-0.01	-0.03	0.07
SCENTinel Score	-0.02	1.00	0.24	0.08	0.88	0.57	0.43
Intensity (>20)	-0.04	0.24	1.00	0.43	0.03	0.14	0.01
Intensity (Cont.)	-0.07	0.08	0.43	1.00	0.00	0.15	0.01
Detection	-0.01	0.88	0.03	0.00	1.00	0.46	0.42
Identification 1	-0.03	0.57	0.14	0.15	0.46	1.00	NA
Identification 2	0.07	0.43	0.01	0.01	0.42	NA	1.00

Agreement statistics

Odor detection

Table 19: Agreement between COVID Result and Odor detection

	NEGATIVE	POSITIVE
Fail	162	6
Pass	696	22

Table 20: Prevalance Adjusted Kappa (95% CI)

Estimate	Lower	Upper
0.585	0.528	0.637

Table 21: Prevalance Adjusted Kappa (Test statistic)

z test statistic	p-value
0.3384	0.36753

Percent agreement: 79.23%
Sensitivity: 0.21
Specificity: 0.81
Negative Predictive Value: 0.97

Odor intensity

Table 22: Agreement between COVID Result and Odor intensity

	NEGATIVE	POSITIVE
	5	0
Fail	9	1
Pass	844	27

Table 23: Prevalance Adjusted Kappa (95% CI)

Estimate	Lower	Upper
0.918	0.888	0.942

Table 24: Prevalance Adjusted Kappa (Test statistic)

z test statistic	p-value
1.2368	0.10808

Percent agreement: 95.37%
Sensitivity: 0.04
Specificity: 0.98
Negative Predictive Value: 0.97

Odor identification

First try at identification only.

Table 25: Agreement between COVID Result and Odor identification

	NEGATIVE	POSITIVE
	5	0
Fail	327	13
Pass	526	15

Table 26: Prevalance Adjusted Kappa (95% CI)

Estimate	Lower	Upper
0.224	0.157	0.288

Table 27: Prevalance Adjusted Kappa (Test statistic)

z test statistic	p-value
0.86562	0.19335

Percent agreement: 60.84%
Sensitivity: 0.46
Specificity: 0.61
Negative Predictive Value: 0.97

SCENTinel Score

Table 28: Agreement between COVID Result and SCENTinel score

	NEGATIVE	POSITIVE
	6	0
Fail	142	6
Pass	710	22

Table 29: Prevalance Adjusted Kappa (95% CI)

Estimate	Lower	Upper
0.627	0.573	0.678

Table 30: Prevalance Adjusted Kappa (Test statistic)

z test statistic	p-value
0.66288	0.2537

Percent agreement: 80.81%
Sensitivity: 0.21
Specificity: 0.83
Negative Predictive Value: 0.97

Logistic regression

```
## logistf(formula = covid_factor ~ odor_discrim_correct + odor_intensity_correct +
##     odor_ident_correct_1, data = mixed_data, na.action = na.exclude,
##     family = binomial)
```

```
##
## Model fitted by Penalized ML
## Coefficients:
##                                coef  se(coef) lower 0.95 upper 0.95       Chisq
## (Intercept)             -1.80288696 0.9369262  -4.121431 -0.1881293 4.906627007
## odor_discrim_correct    -0.03610571 0.5013454  -1.005880  1.0218777 0.005004282
## odor_intensity_correct -1.38203413 0.9175748  -2.972127  0.9024995 1.685771803
## odor_ident_correct_1    -0.30077344 0.4283441  -1.133502  0.5796637 0.472015114
##                                  p method
## (Intercept)             0.02675384      2
## odor_discrim_correct    0.94360393      2
## odor_intensity_correct 0.19415928      2
## odor_ident_correct_1    0.49206158      2
##
## Method: 1-Wald, 2-Profile penalized log-likelihood, 3-None
##
## Likelihood ratio test=2.805763 on 3 df, p=0.422552, n=880
## Wald test = 319.2032 on 3 df, p = 0

## [1] "AIC: 244.11"

## [1] "McF pseudo-R: 0.0116"

## logistf(formula = covid_factor ~ odor_discrim_correct + odor_intensity +
##     odor_ident_correct_1, data = mixed_data, na.action = na.exclude,
##     family = binomial)
##
## Model fitted by Penalized ML
## Coefficients:
##                              coef    se(coef)  lower 0.95   upper 0.95
## (Intercept)          -1.69882165 0.731963263 -3.29831385 -0.340185340
## odor_discrim_correct -0.11274935 0.499572720 -1.07628058  0.945089995
## odor_intensity       -0.01939905 0.008885231 -0.03661449 -0.000577802
## odor_ident_correct_1 -0.18782969 0.426932243 -1.02699183  0.687989495
##                           Chisq          p method
## (Intercept)          6.20676652 0.01272629      2
## odor_discrim_correct 0.04865995 0.82541168      2
## odor_intensity       4.06716808 0.04372452      2
## odor_ident_correct_1 0.18514728 0.66698656      2
##
## Method: 1-Wald, 2-Profile penalized log-likelihood, 3-None
##
## Likelihood ratio test=5.035219 on 3 df, p=0.1692363, n=880
## Wald test = 313.0089 on 3 df, p = 0

## [1] "AIC: 231.68"

## [1] "McF pseudo-R: 0.0216"
```

Delta dominant sample

SARS-CoV-2 results

Table 31: SCV-2 infection

	n
NEGATIVE	678
POSITIVE	28

Symptoms

Table 32: Symptoms

	NEGATIVE	POSITIVE	p	test
n	678	28		
Fever or chills = 1 (%)	90 (13.3)	14 (50.0)	<0.001	
Cough = 1 (%)	48 (7.1)	4 (14.3)	0.289	
Shortness of breath = 1 (%)	60 (8.8)	13 (46.4)	<0.001	
Fatigue = 1 (%)	114 (16.8)	11 (39.3)	0.005	
Muscle or body aches = 1 (%)	51 (7.5)	12 (42.9)	<0.001	
Headache = 1 (%)	20 (2.9)	6 (21.4)	<0.001	
New loss of taste/smell = 1 (%)	62 (9.1)	10 (35.7)	<0.001	
Sore throat = 1 (%)	47 (6.9)	6 (21.4)	0.013	
Congestion/runny nose = 1 (%)	15 (2.2)	4 (14.3)	0.001	
Nausea or vomiting = 1 (%)	13 (1.9)	16 (57.1)	<0.001	
Diarrhea = 1 (%)	13 (1.9)	4 (14.3)	<0.001	
None = 1 (%)	481 (70.9)	2 (7.1)	<0.001	

Correlations

	SARS-CoV-2+	SCENTinel™ Score	Intensity (>20)	Intensity (Cont.)	Detection	Identification 1	Identification 2
SARS-CoV-2+	1.00	-0.26	-0.47	-0.34	-0.11	-0.06	-0.22
SCENTinel™ Score	-0.26	1.00	0.61	0.38	0.67	0.37	0.37
Intensity (>20)	-0.47	0.61	1.00	0.58	0.19	0.16	0.22
Intensity (Cont.)	-0.34	0.38	0.58	1.00	0.15	0.15	0.19
Detection	-0.11	0.67	0.19	0.15	1.00	0.26	0.35
Identification 1	-0.06	0.37	0.16	0.15	0.26	1.00	NA
Identification 2	-0.22	0.37	0.22	0.19	0.35	NA	1.00

Agreement statistics

Odor detection

Table 34: Agreement between COVID Result and Odor detection

	NEGATIVE	POSITIVE
Fail	33	5
Pass	645	23

Table 35: Prevalance Adjusted Kappa (95% CI)

Estimate	Lower	Upper
0.841	0.796	0.879

Table 36: Prevalance Adjusted Kappa (Test statistic)

z test statistic	p-value
2.98484	0.00142

Percent agreement: 92.07%
Sensitivity: 0.18
Specificity: 0.95
Negative Predictive Value: 0.97

Odor intensity

Table 37: Agreement between COVID Result and Odor intensity

	NEGATIVE	POSITIVE
	1	0
Fail	5	10
Pass	672	18

Table 38: Prevalance Adjusted Kappa (95% CI)

Estimate	Lower	Upper
0.935	0.903	0.958

Table 39: Prevalance Adjusted Kappa (Test statistic)

z test statistic	p-value
12.56795	0

Percent agreement: 96.6%
Sensitivity: 0.36
Specificity: 0.99
Negative Predictive Value: 0.97

Odor identification

First try at identification only.

Table 40: Agreement between COVID Result and Odor identification

	NEGATIVE	POSITIVE
	7	3
Fail	129	8
Pass	542	17

Table 41: Prevalance Adjusted Kappa (95% CI)

Estimate	Lower	Upper
0.58	0.516	0.64

Table 42: Prevalance Adjusted Kappa (Test statistic)

z test statistic	p-value
1.57735	0.05736

Percent agreement: 77.9%
Sensitivity: 0.29
Specificity: 0.8
Negative Predictive Value: 0.97

SCENTinel Score

Table 43: Agreement between COVID Result and SCENTinel score

	NEGATIVE	POSITIVE
	9	3
Fail	23	8
Pass	646	17

Table 44: Prevalance Adjusted Kappa (95% CI)

Estimate	Lower	Upper
0.885	0.845	0.917

Table 45: Prevalance Adjusted Kappa (Test statistic)

z test statistic	p-value
6.78757	0

Percent agreement: 92.63%
Sensitivity: 0.29
Specificity: 0.95
Negative Predictive Value: 0.97

Logistic regression

```
## logistf(formula = covid_factor ~ odor_discrim_correct + odor_intensity_correct +
##     odor_ident_correct_1, data = delta_data, na.action = na.exclude,
##     family = binomial)
##
## Model fitted by Penalized ML
## Coefficients:
##                              coef  se(coef) lower 0.95 upper 0.95       Chisq
## (Intercept)            0.72552358 0.7332078 -0.7513171  2.2486394  0.94539701
## odor_discrim_correct   -0.66506170 0.7311645 -2.0298170  0.9817814  0.70365276
## odor_intensity_correct -3.70529224 0.6538360 -5.0503194 -2.4285601 28.31642963
## odor_ident_correct_1    0.07117006 0.5549003 -0.9598345  1.2945394  0.01596842
##                                   p method
## (Intercept)            3.308937e-01      2
## odor_discrim_correct   4.015590e-01      2
## odor_intensity_correct 1.030179e-07      2
## odor_ident_correct_1   8.994420e-01      2
##
## Method: 1-Wald, 2-Profile penalized log-likelihood, 3-None
##
## Likelihood ratio test=35.02371 on 3 df, p=1.204279e-07, n=696
## Wald test = 234.6028 on 3 df, p = 0
```

```
## [1] "AIC: 180.57"
```

```
## [1] "McF pseudo-R: 0.1655"
```

```
## logistf(formula = covid_factor ~ odor_discrim_correct + odor_intensity +
##     odor_ident_correct_1, data = delta_data, na.action = na.exclude,
##     family = binomial)
##
## Model fitted by Penalized ML
## Coefficients:
##                             coef    se(coef)  lower 0.95  upper 0.95
## (Intercept)           0.598509323 0.687242313 -0.81321443  1.98055095
## odor_discrim_correct -0.590799107 0.716953199 -1.94456410  0.98568954
## odor_intensity       -0.046545899 0.008075673 -0.06292397 -0.03058333
## odor_ident_correct_1  0.001076368 0.528026711 -0.99233429  1.14745354
##                             Chisq            p method
## (Intercept)          7.242510e-01 3.947530e-01      2
```

```
## odor_discrim_correct 5.961355e-01 4.400563e-01      2
## odor_intensity       3.054501e+01 3.262106e-08      2
## odor_ident_correct_1 3.988521e-06 9.984065e-01      2
##
## Method: 1-Wald, 2-Profile penalized log-likelihood, 3-None
##
## Likelihood ratio test=36.86994 on 3 df, p=4.902354e-08, n=696
## Wald test = 212.5098 on 3 df, p = 0
```

```
## [1] "AIC: 168.92"
```

```
## [1] "McF pseudo-R: 0.1827"
```

Omicron dominant sample

SARS-CoV-2 results

Table 46: SCV-2 infection

	n
NEGATIVE	316
POSITIVE	71

Symptoms

Table 47: Symptoms

	NEGATIVE	POSITIVE	p	test
n	316	71		
Fever or chills = 1 (%)	46 (14.6)	40 (56.3)	<0.001	
Cough = 1 (%)	28 (8.9)	21 (29.6)	<0.001	
Shortness of breath = 1 (%)	28 (8.9)	42 (59.2)	<0.001	
Fatigue = 1 (%)	59 (18.7)	45 (63.4)	<0.001	
Muscle or body aches = 1 (%)	21 (6.6)	20 (28.2)	<0.001	
Headache = 1 (%)	7 (2.2)	9 (12.7)	<0.001	
New loss of taste/smell = 1 (%)	22 (7.0)	22 (31.0)	<0.001	
Sore throat = 1 (%)	18 (5.7)	4 (5.6)	1.000	
Congestion/runny nose = 1 (%)	4 (1.3)	9 (12.7)	<0.001	
Nausea or vomiting = 1 (%)	4 (1.3)	4 (5.6)	0.061	
Diarrhea = 1 (%)	5 (1.6)	6 (8.5)	0.006	
None = 1 (%)	220 (69.6)	7 (9.9)	<0.001	

Correlations

	SARS-CoV-2+	SCENTinel Score	Intensity (>20)	Intensity (Cont.)	Detection	Identification 1	Identification 2
SARS-CoV-2+	1.00	0.00	0.03	-0.08	0.03	-0.07	-0.01
SCENTinel Score	0.00	1.00	0.42	0.13	0.77	0.33	0.25
Intensity (>20)	0.03	0.42	1.00	0.28	0.17	0.06	0.12
Intensity (Cont.)	-0.08	0.13	0.28	1.00	0.09	0.12	-0.20
Detection	0.03	0.77	0.17	0.09	1.00	0.25	0.25
Identification 1	-0.07	0.33	0.06	0.12	0.25	1.00	NA
Identification 2	-0.01	0.25	0.12	-0.20	0.25	NA	1.00

Agreement statistics

Odor detection

Table 49: Agreement between COVID Result and Odor detection

	NEGATIVE	POSITIVE
Fail	14	2
Pass	302	69

Table 50: Prevalance Adjusted Kappa (95% CI)

Estimate	Lower	Upper
0.571	0.482	0.651

Table 51: Prevalance Adjusted Kappa (Test statistic)

z test statistic	p-value
-0.61708	0.73141

Percent agreement: 78.55%
Sensitivity: 0.03
Specificity: 0.96
Negative Predictive Value: 0.81

Odor intensity

Table 52: Agreement between COVID Result and Odor intensity

	NEGATIVE	POSITIVE
Fail	2	0
Pass	314	71

Table 53: Prevalance Adjusted Kappa (95% CI)

Estimate	Lower	Upper
0.623	0.538	0.698

Table 54: Prevalance Adjusted Kappa (Test statistic)

z test statistic	p-value
-0.67209	0.74924

Percent agreement: 81.14%
Sensitivity: 0
Specificity: 0.99
Negative Predictive Value: 0.82

Odor identification

First try at identification only.

Table 55: Agreement between COVID Result and Odor identification

	NEGATIVE	POSITIVE
	5	0
Fail	54	17
Pass	257	54

Table 56: Prevalance Adjusted Kappa (95% CI)

Estimate	Lower	Upper
0.435	0.338	0.524

Table 57: Prevalance Adjusted Kappa (Test statistic)

z test statistic	p-value
1.28611	0.0992

Percent agreement: 70.8%
Sensitivity: 0.24
Specificity: 0.81
Negative Predictive Value: 0.83

SCENTinel Score

Table 58: Agreement between COVID Result and SCENTinel score

	NEGATIVE	POSITIVE
	6	1
Fail	9	2
Pass	301	68

Table 59: Prevalance Adjusted Kappa (95% CI)

Estimate	Lower	Upper
0.595	0.507	0.673

Table 60: Prevalance Adjusted Kappa (Test statistic)

z test statistic	p-value
-0.02077	0.50829

Percent agreement: 78.29%
Sensitivity: 0.03
Specificity: 0.95
Negative Predictive Value: 0.82

Logistic regression

```
## logistf(formula = covid_factor ~ odor_discrim_correct + odor_intensity_correct +
##     odor_ident_correct_1, data = omi_data, na.action = na.exclude,
##     family = binomial)
##
## Model fitted by Penalized ML
## Coefficients:
##                               coef  se(coef) lower 0.95 upper 0.95       Chisq
## (Intercept)            -1.53861784 1.5618875 -6.4858091  1.0374150 1.252007239
## odor_discrim_correct    0.40135390 0.7103470 -0.8756961  2.0642863 0.331911120
## odor_intensity_correct  0.08163015 1.5658719 -2.5112461  5.0387960 0.002697876
## odor_ident_correct_1   -0.48507585 0.3192776 -1.0997985  0.1625115 2.192140293
##                                p method
## (Intercept)            0.2631695      2
## odor_discrim_correct   0.5645359      2
## odor_intensity_correct 0.9585756      2
## odor_ident_correct_1   0.1387164      2
##
## Method: 1-Wald, 2-Profile penalized log-likelihood, 3-None
##
## Likelihood ratio test=2.248966 on 3 df, p=0.522368, n=382
## Wald test = 125.1898 on 3 df, p = 0
```

```
## [1] "AIC: 362.86"

## [1] "McF pseudo-R: 0.0062"

## logistf(formula = covid_factor ~ odor_discrim_correct + odor_intensity +
##     odor_ident_correct_1, data = omi_data, na.action = na.exclude,
##     family = binomial)
##
## Model fitted by Penalized ML
## Coefficients:
##                             coef    se(coef)  lower 0.95  upper 0.95     Chisq
## (Intercept)          -0.71399400 0.891866458 -2.65319987 0.947237016 0.6720199
## odor_discrim_correct  0.53405703 0.722590754 -0.74497261 2.215420747 0.5948529
## odor_intensity       -0.01156221 0.008090304 -0.02737535 0.004636756 1.9782093
## odor_ident_correct_1 -0.43632553 0.321894137 -1.05609238 0.217352415 1.7504199
##                              p method
## (Intercept)          0.4123483      2
## odor_discrim_correct 0.4405486      2
## odor_intensity       0.1595792      2
## odor_ident_correct_1 0.1858240      2
##
## Method: 1-Wald, 2-Profile penalized log-likelihood, 3-None
##
## Likelihood ratio test=4.270278 on 3 df, p=0.2337192, n=382
## Wald test = 124.889 on 3 df, p = 0

## [1] "AIC: 350.09"

## [1] "McF pseudo-R: 0.0122"
```

Odor intensity plus symptoms - Delta period

SARS-CoV-2+ ~ symptoms

```
## logistf(formula = covid_factor ~ symptoms, data = moddf, na.action = na.exclude,
##     family = binomial)
##
## Model fitted by Penalized ML
## Coefficients:
##                   coef   se(coef) lower 0.95 upper 0.95   Chisq            p
## (Intercept) -4.3563047 0.33999658 -5.0918754 -3.7449151     Inf 0.000000e+00
## symptoms     0.5638369 0.08288943  0.4046201  0.7335511 45.7559 1.339451e-11
##             method
## (Intercept)      2
## symptoms         2
##
## Method: 1-Wald, 2-Profile penalized log-likelihood, 3-None
##
## Likelihood ratio test=45.75591 on 1 df, p=1.339451e-11, n=694
## Wald test = 188.3649 on 1 df, p = 0

## [1] "AIC: 166.1"
```

```
## [1] "McF pseudo-R: 0.2201"
```

SARS-CoV-2+ ~ symptoms + intensity (cont.)

```
## logistf(formula = covid_factor ~ symptoms + intensity_r, data = moddf,
##     na.action = na.exclude, family = binomial)
##
## Model fitted by Penalized ML
## Coefficients:
##                    coef    se(coef)  lower 0.95  upper 0.95     Chisq
## (Intercept) -1.55966540 0.684335759 -2.97872149 -0.23883644  5.403999
## symptoms     0.47630182 0.089822182  0.29958456  0.65869216 26.611991
## intensity_r  0.03618081 0.008622621  0.01910448  0.05367756 16.970447
##                        p method
## (Intercept) 2.009066e-02      2
## symptoms    2.486954e-07      2
## intensity_r 3.796622e-05      2
##
## Method: 1-Wald, 2-Profile penalized log-likelihood, 3-None
##
## Likelihood ratio test=63.11538 on 2 df, p=1.976197e-14, n=694
## Wald test = 177.4771 on 2 df, p = 0

## [1] "AIC: 139.84"

## [1] "McF pseudo-R: 0.3172"

## [1] "LL MOD 0:-81.05"

## [1] "LL MOD 1:-67.92"

## [1] "Test stat:26.26"

## [1] "P-value:2.985e-07"
```

SARS-CoV-2+ ~ symptoms + intensity (dichotomous)

```
## logistf(formula = covid_factor ~ symptoms + intensity_dich_r,
##     data = moddf, na.action = na.exclude, family = binomial)
##
## Model fitted by Penalized ML
## Coefficients:
##                       coef   se(coef) lower 0.95 upper 0.95    Chisq
## (Intercept)      -4.409287 0.34992280 -5.1711167 -3.7811105      Inf
## symptoms          0.494364 0.08959921  0.3179454  0.6754512 27.81783
## intensity_dich_r  2.886996 0.70422150  1.4727271  4.3283830 15.22902
##                             p method
## (Intercept)      0.000000e+00      2
## symptoms         1.332917e-07      2
## intensity_dich_r 9.522853e-05      2
##
```

```
## Method: 1-Wald, 2-Profile penalized log-likelihood, 3-None
##
## Likelihood ratio test=62.07477 on 2 df, p=3.319567e-14, n=694
## Wald test = 185.8958 on 2 df, p = 0
```

```
## [1] "AIC: 150.69"
```

```
## [1] "McF pseudo-R: 0.2973"
```

```
## [1] "LL MOD 0:-81.05"
```

```
## [1] "LL MOD 2:-73.35"
```

```
## [1] "Test stat:15.41"
```

```
## [1] "P-value:8.67387e-05"
```

Odor intensity effect size - Delta dominant period

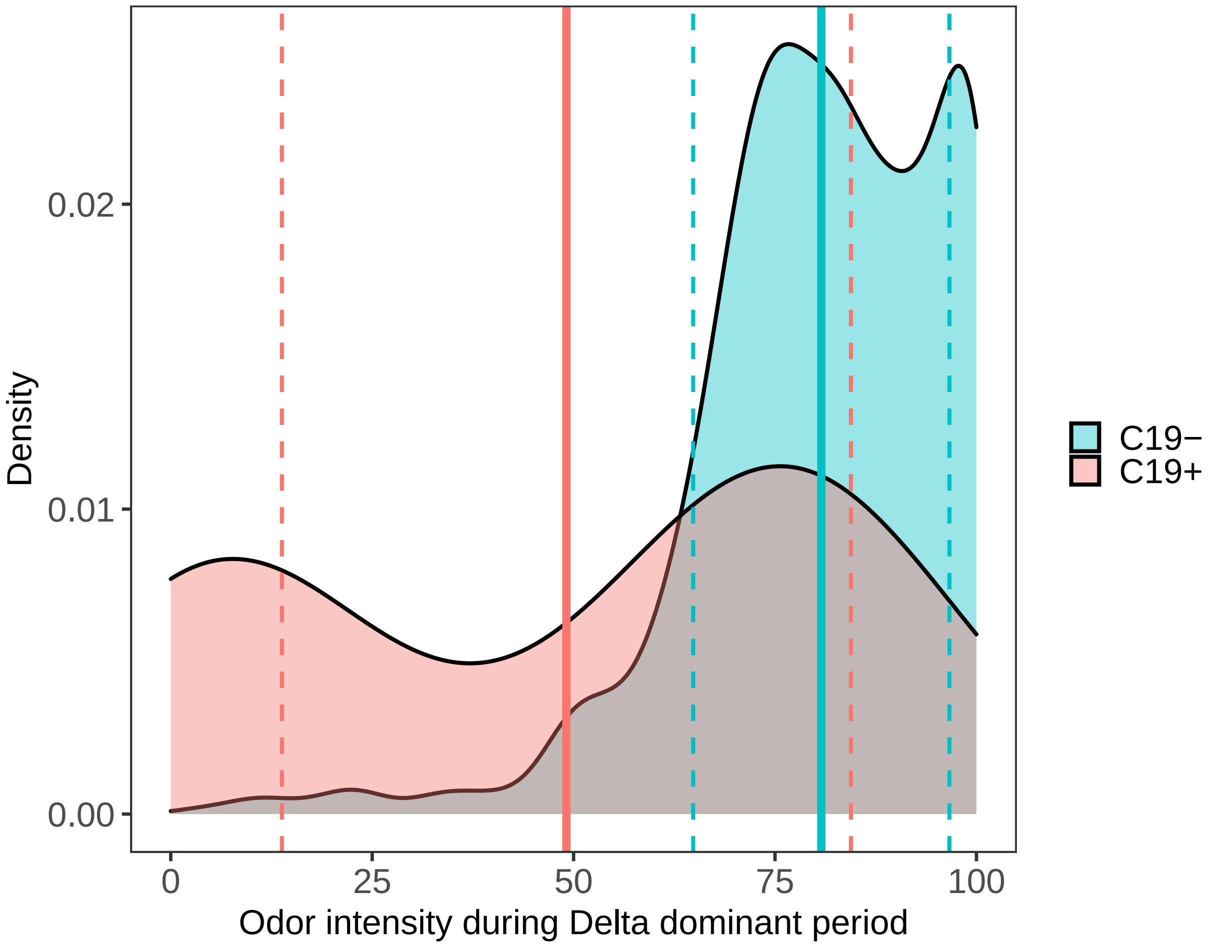

Cohens d: 1.16
