# Supplementary material for: Using SCENTinel® to predict SARS-CoV-2 infection: insights from a community sample during dominance of Delta and Omicron variants
Source: Front Public Health. 2024 Apr 10;12:1322797. doi: 10.3389/fpubh.2024.1322797 (PMC11041634; doi:10.3389/fpubh.2024.1322797)
Supplement: Supplementary Appendix S2 — All pre-registered analyses. [file Data_Sheet_7.PDF]

S2 Figure. SCENTinel® Survey Questions.

Start by pointing your camera at the QR code, located on the SCENTinel card. Troubles with the QR code? Go to redcap.link/SCENTinel02. Code: 111000

On the back of your SCENTinel card, there are three boxes: A, B, and C.

From the bottom left corner, peel up (but do not remove) the box labeled A.

Smell odor A and then close it.

Repeat with odor B.

Repeat with odor C.

Which odor smells the *STRONGEST*?

○ A
○ B
○ C

Now rate the INTENSITY of the odor.

(To rate the intensity, click on the slider first. Then move the slider.)

No Smell — Very Strong Smell

50

reset

What does the odor smell like?

○ Flower

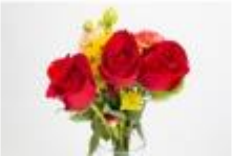

○ Popcorn

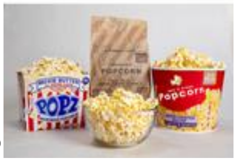

○ Orange

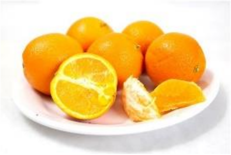

○ Chocolate

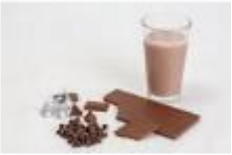

Branching logic: if correct answer, then go to end of survey.

S2 Figure. SCENTinel® Survey Questions.

You selected CHOCOLATE which was incorrect. Try again!

○ Flower

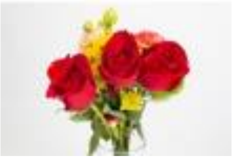

○ Popcorn

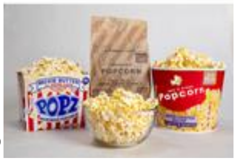

○ Orange

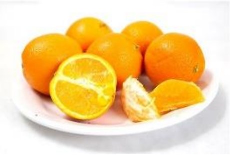

Thank you for completing the SCENTinel Rapid Smell Test today.

You may now dispose of your SCENTinel test, as it cannot be used again for this study.
